# Three *BnaIAA7* homologs are involved in auxin/brassinosteroid-mediated plant morphogenesis in rapeseed (*Brassica napus L.*)

**DOI:** 10.1007/s00299-019-02410-4

**Published:** 2019-04-22

**Authors:** Ming Zheng, Maolong Hu, Hongli Yang, Min Tang, Liang Zhang, Hongfang Liu, Xiaokang Li, Jinglin Liu, Xingchao Sun, Shihang Fan, Jiefu Zhang, William Terzaghi, Huiming Pu, Wei Hua

**Affiliations:** 1 Oil Crops Research Institute of the Chinese Academy of Agricultural Sciences, Key Laboratory of Biology and Genetic Improvement of Oil Crops, Ministry of Agriculture, Wuhan, 430062 China; 2 Key Laboratory of Cotton and Rapeseed, Ministry of Agriculture, Institute of Industrial Crops, Jiangsu Academy of Agricultural Sciences, Nanjing, 210014 China; 3 Department of Biology, Wilkes University, Wilkes-Barre, PA 18766 USA

**Keywords:** *Brassica napus*, Plant height, Auxin, Brassinosteroid, *BnaIAA7*s, *BnaARF8*, *BnaBZR1*

## Abstract

**Key message:**

*BnaIAA7* crosstalk with BR signaling is mediated by the interaction between *BnaARF8* and *BnaBZR1* to regulate rapeseed plant morphogenesis.

**Abstract:**

Auxin (indole-3-acetic acid, IAA) and brassinosteroids (BRs) are essential regulators of plant morphogenesis. However, their roles in rapeseed have not been reported. Here, we identified an *extremely dwarf1* (*ed1*) mutant of rapeseed that displays reduced stature, short hypocotyls, as well as wavy and curled leaves. We isolated *ED1* by map-based cloning, and found that it encodes a protein homologous to AtIAA7. *ED1* acts as a repressor of IAA signaling, and IAA induces its degradation through its degron motif. A genomic-synteny analysis revealed that *ED1* has four homologs in rapeseed, but two were not expressed. Analyses of transcriptomes and of various mutant BnaIAA7s in transgenic plants revealed that the three expressed *BnaIAA7* homologs had diverse expression patterns. *ED1*/*BnaC05*.*IAA7* predominantly functioned in stem elongation, *BnaA05.IAA7* was essential for reproduction, while *BnaA03.IAA7* had the potential to reduce plant height. Physical interaction assays revealed that the three BnaIAA7 homologs interacted in different ways with BnaTIRs/AFBs and BnaARFs, which may regulate the development of specific organs. Furthermore, BnaARF8 could directly interact with the BnaIAA7s and BnaBZR1. We propose that *BnaIAA7*s interact with BR signaling via *BnaARF8* and *BnaBZR1* to regulate stem elongation in rapeseed.

**Electronic supplementary material:**

The online version of this article (10.1007/s00299-019-02410-4) contains supplementary material, which is available to authorized users.

## Introduction

Plant height (PH) is under the integrated control of plant hormones and environmental factors (Salas Fernandez et al. 2009). Among the former, the brassinosteroid (BRs) and gibberellin (GAs) biosynthetic and signaling pathways are the crucial control factors, and mutants with defects in these processes display dwarf phenotypes (Sun 2008; Clouse 2011). In addition, BRs are also involved in the regulation of root growth and the morphological development of leaves and flowers (Zhu et al. 2013). The strigolactones and auxins are other families of plant hormones that affect PH and the outgrowth of axillary buds (Zhao 2010; Ruyter-Spira et al. 2013). Although these mechanisms are well-established in model species (Arabidopsis and rice), the identities of these signals are not known in rapeseed.

The plant growth regulator auxin (indole-3-acetic acid, IAA) plays a crucial role in plant growth and development, including lateral root formation, stem elongation, apical dominance, gravitropism, and vascular development (Woodward and Bartel 2005). Active auxins are synthesized in shoot apical meristems, young leaves, and root meristems (Ljung et al. 2001), and are then transported in or out of the cell by auxin carriers, such as AUX1, PINs, and ABCB/PGPs (Blakeslee et al. 2005). After decades of work, an auxin-signaling pathway has been elucidated (Gray et al. 2001; Dharmasiri et al. 2005; Wang and Estelle 2014). Auxin signal transduction begins with specific binding of auxin receptors TRANSPORT INHIBITOR RESPONSE 1/AUX SIGNALING F-BOX PROTEINS (TIR1/AFBs), and Auxin/Indole-Acetic Acid (Aux/IAAs) transcriptional repressors (Dharmasiri et al. 2005; Calderon Villalobos et al. 2012; Salehin et al. 2015). The Auxin-Response Factors (ARFs) are the downstream targets of Aux/IAAs that bind auxin-response elements (AuxRE) in the promoters of auxin-response genes to regulate their transcription (Weijers et al. 2005).

Most Aux/IAA repressor proteins have four conserved domains (I–IV) (Abel et al. 1996). Domain I is an L × L×L motif, with a transcriptional repression activity, and it recruits the co-repressor protein, TOPLESS (TPL), to inactivate the ARFs (Tiwari et al. 2004). The C-terminal domain (III/IV) is required for homo-dimerization between Aux/IAA repressors or hetero-dimerization between Aux/IAAs and ARF regulators (Kim et al. 1997; Ulmasov et al. 1999). Domain II is related to the instability of Aux/IAA repressors in the presence of auxin, and it can directly interact with TIR1 and auxin (Worley et al. 2000). A mutation of the conserved motif (GWPPV) in domain II abolishes the interactions between TIR1/AFBs and Aux/IAAs, which prevents their degradation and thus alters the auxin response (Ramos et al. 2001; Dharmasiri et al. 2005). In Arabidopsis, dominant missense mutations in domain II produce a series of gain-of-function mutants, such as *shy2/iaa3*, *axr2/iaa7*, *bdl/iaa12*, *slr/iaa14*, *axr3/iaa17*, *msg2/iaa19*, and *iar2/iaa28*; these mutants display diverse defects in growth and development, including slowed root growth, few lateral roots, curled leaves, dwarfism, and decreased gravitropism (Nagpal et al. 2000; Reed 2001). Conversely, most of the single copy loss-of-function *ARF* mutants fail to show any obvious growth defects, except for *hss/arf2*, *ett/arf3*, *mp/arf5*, and *nph4/arf7* (Hardtke and Berleth 1998; Harper et al. 2000; Nemhauser et al. 2000). Double *ARF* mutants also display strong auxin phenotypes, suggesting that there are unique and overlapping functions among the *ARF*s in Arabidopsis (Nagpal et al. 2005; Okushima et al. 2005).

The two key transcription factors in the BR signaling pathway are BRASSINAZOLE-RESISTANT 1 (BZR1) and BRI1-EMSSUPPRESSOR (BES1). They positively mediate BR responses depending on their phosphorylation status (Sun et al. 2010). Recently, auxin and BR were reported to collaborate during some developmental processes (Wang et al. 2014). In Arabidopsis, ARF6 interacts with PIF4 and BZR1 to cooperatively regulate Arabidopsis hypocotyl elongation (Oh et al. 2014). In addition, ARF5 recruits BES1 to its target promoter when treated with IAA or BR (Walcher and Nemhauser 2012). BIN2 can directly phosphorylate ARF2 and ARF7, and, therefore, mediate hypocotyl elongation and lateral root development, respectively (Vert et al. 2008). In rice (*Oryza sativa*), *OsARF19* controls leaf angles by positively regulating *OsBRI1* expression (Zhang et al. 2015). Despite these significant advances, mutations in auxin-related genes have not yet been identified in rapeseed, which is allopolyploid. Therefore, the overall understanding of the molecular mechanisms controlling auxin- or BR-mediated plant growth and development remains limited in allopolyploid plants.

In this study, we identified a rapeseed semi-dominant gene *extremely dwarf1* (*ED1*) that encoded an Aux/IAA7 protein, which acts as a transcriptional repressor in auxin signal transduction. In addition, the rapeseed genome has four other *BnaIAA7* homologs. These *BnaIAA7* homologs have redundant but divergent functions in rapeseed plant morphogenesis, where *ED1*/*BnaC05.IAA7* predominantly functions in stem elongation. Furthermore, we found that BnaARF8 directly interacts with all three expressed BnaIAA7s and BnaBZR1. The rapeseed BnaIAA7–BnaARF8–BnaBZR1 interaction model enhances our understanding of the ways in which BnaIAA7 proteins participate in BR-mediated growth responses.

## Materials and methods

### Plant materials and growth

The *ed1* mutant is a spontaneous mutant obtained from the field and back-crossed with the Ningyou 336 (NY336) strain to generate a homozygous line (from BC_5_F_2_) in the ‘NY336’ background. The wild-type (WT) rapeseed varieties used in this study were ‘NY336’ (semi-winter-type) and ‘862’ (spring-type) for transformation analyses. The rapeseed F_2_ population used for genetic mapping was grown in its natural growing season in Wuhan, China. The seedlings of WT, mutants, and transgenic plants were grown in the greenhouse (under 16 h of light/8 h of dark at 20–23 °C).

### Histological analysis

For microscopy, rapeseed stem (five-leaf stage) and young leaf (three-leaf stage) segments were fixed in FAA (formalin–acetic acid–alcohol) solution overnight, followed by a series of dehydration and infiltration steps. The samples were then embedded in Paraffin Plus (Thermo Fisher). The tissues were sliced to reach a thickness of 8–10 μm (Leica RM2265) and then stained with 0.05% toluidine blue. These were observed under an Eclipse E80i light microscope (Nikon). The stem cell size and number were calculated using the Image J software.

The rapeseed leaves were cleaned with chloral hydrate solution (200 g of chloral hydrate, 20 g of glycerol, and 50 ml of dH_2_O) and photographed with a digital camera (Nikon).

### Analysis of endogenous IAA and BR content

For IAA (total) and BR (24-epiBL and 24-epiCS) measurements, 200 mg or 1 g fresh leaves were ground to a fine powder in liquid nitrogen, and the samples were extracted, purified, and analyzed following the standard procedure for liquid chromatography–mass spectrometry (LC–MS) as previously described (Xin et al. 2013; Wang et al. 2015). Each sample was analyzed in triplicate.

### Map-based cloning and complementation analysis

To map and identify the *ED1* gene, the *ed1* mutant was crossed with Y96 (*Brassica napus*) to generate the F_2_ population. Ninety-two individuals from the F_2_ population were genotyped using a 60-k SNP array for preliminary mapping. 750 F_2_ plants with normal phenotypes were then used for fine mapping with SNP markers (Supplementary Table 1). To confirm that mutation of *ED1* resulted in the *ed1* phenotype, we generated the *Pro*_*35S*_*::ed1* and *Pro*_*ED1*_*::ed1* constructs and introduced them into the hypocotyls of a WT variety ‘862’ (spring-type) via *Agrobacterium*-mediated transformation (Zhou et al. 2002).

### qRT-PCR

Total RNA was extracted using RNA Prep Pure Plant Kits (Tiangen). The cDNA was synthesized using the First cDNA Transcriptase and Oligo (dT)18 primer (Takara). qRT-PCR was performed using a Fast Start Universal Probe Master Mix (Roche) in an ABI 7500 Fast PCR system with three biological replicates. The rapeseed *TMA7* gene (*BnaC05g11560D*) was used as control. Primers for qRT-PCR are listed in Supplemental Table 6. Data were analyzed following the relative quantification method ($2^{{ - \Delta \Delta C_{T} }}$).

### *Pro*_*BnaIAA7s*_*:: GUS* reporter gene construction and GUS staining

The fragments’ upstream of the three BnaIAA7s ATG start codons (1967, 2387, and 1970 bp for *ED1*, *BnaA03.IAA7,* and *BnaA05.IAA7*, respectively) were cloned into the DX2181 vector to generate *Pro*_*C05*_*::GUS*, *Pro*_*A03*_*::GUS*, and *Pro*_*A05*_*::GUS*. Primers for PCR amplification are listed in Supplemental Table 6. The three plasmids were transformed into 862 hypocotyls and were then selected using hygromycin (15 mg L^−1^). Two independent T_2_ positive transgenic lines were used for histochemical analysis, where GUS staining was performed, as described by Zheng et al. (2015).

### Transient expression assays

The *ED1* full-length cDNA was cloned into the vectors, pCAMBIA-1305 or pGreenII 0800-LUC, to generate GFP-ED1, ED1-LUC, and ed1-LUC plasmids for subcellular localization analysis or auxin-mediated degradation assays, respectively. The GFP-ED1 fusion proteins were transiently expressed in epidermal cells of *Nicotiana benthamiana* leaves, where the FIB2-mCherry was used as a nuclear marker (Zheng et al. 2016). The rapeseed protoplast isolation and transformation procedure was carried out as described by Zheng et al. (2018). For measurement of the luciferase activity, rapeseed protoplasts were transformed with ED1/ed1-LUC plasmids and incubated for 18 h; they were then treated with or without 1 or 10 μM IAA for 1 or 4 h, respectively. 40 μM MG132 was added to control samples and incubated for 1 h before IAA treatment to inhibit proteasomal degradation of ED1. The ratios of firefly luciferase (fLUC)/Renilla luciferase (rLUC) were detected using the Dual-Luciferase Reporter Assay System (Promega).

### Generating defined mutations in *BnaA03.IAA7* and *BnaA05.IAA7*

To determine whether the other *BnaIAA7* copies have a similar function in auxin signal transduction, we constructed *Pro*_*A03*_*::A03*D (P86L) and *Pro*_*A05*_*::A05D* (P87L) that had the same amino acid substitution (P–L) in their GWPPV motifs as *ed1*. Full-length *ed1* cDNA was then cloned into the binary vector under control of the *BnaA03.IAA7* promoter to generate *Pro*_*A03*_*::ed1*. The three plasmids were transformed into ‘862’ hypocotyls and selected using phosphinothricin (10 mg L^−1^). Two independent T_2_ positive transgenic lines of each construct were used in further phenotypic observation.

### Y2H assay

For BnaTIRs/AFBs interaction assays, *ED1*, *ed1*, *BnaA03.IAA7*, and *BnaA05.IAA7* full-length cDNAs were cloned into pB424AD (Clontech), while *BnaTIRs/AFBs* full-length cDNA were cloned into pLexA (Clontech). *BnaIAA7/TIRs* or *AFBs* were transformed into the EGY48A strain and selected on control media plates (SD/−Ura/−His/−Trp) for 3 days at 30 °C. Three single clones were then selected by spotting on a selective plate (SD/Gal/Raff/−Ura–His–Trp + X–Gal + BU salts) by incubation for 3 days. For BnaIAA7 and BnaARF interaction assays, the three *BnaIAA7* full-length cDNAs were cloned into pGBKT7 (Clontech), and copies of *BnaARF* full-length cDNA were cloned into pGADT7 (Clontech). The *BnaIAA7/ARF* plasmids were then transformed into AH109 strain and selected on control media (SD/–Leu–Trp) plate by incubation for 3 days at 30 °C. The interactions were detected on the selective plate (SD/−Leu−Trp−His−Ade + X-a−Gal). Primers for PCR amplification are listed in Supplementary Table 6.

### Bimolecular fluorescence complementation (BiFC) assay

The full-length cDNA of *BnaBZR1* was cloned into the pVYN (Venus) vector to construct the NV-BnaBZR1 fusion protein and *BnaARF8* cloned into the pVYC (Venus) vector was used to produce CV-BnaARF8 fusion proteins, respectively (primer sequences are listed in Supplementary Table 6). The BiFC analyses were performed in rapeseed protoplasts, and NV-bZIP63/CV-bZIP63 was used as positive control (Walter et al. 2004).

### RNA-Seq analysis

Total RNA was extracted from 4-week-old 862 strain, *35S*::*ed1* and *Pro*_*ED1*_::*ed1,* leaves with two biological replicates using RNA Prep Pure Plant Kits (Tiangen). The libraries were constructed and sequenced using an Illumina HiSEquation 2000 (Benagen). ~ 25 million raw reads from each sample were collected and filtered. Clean reads were mapped to the reference genome (http://www.genoscope.cns.fr/brassicanapus/) using Hisat2. The gene expression levels were calculated by the FPKM method based on the number of uniquely mapped reads. Differential expression analysis was performed with the DESeq2 R package using |log2 (fold change)| ≥ 1 and a corrected *P* < 0.05 as the threshold for significant differential expression. *P* values were adjusted using the Benjamini and Hochberg approach to control for false discovery rates. Functional enrichment analyses, including Gene Ontology (GO) and KEGG, were performed on DEGs, which were compared to the whole genome background using the hypergeometric test with Benjamini and Hochberg’s false discovery rate correction at the significance threshold of 0.05.

## Results

### *ed1* is a semi-dominant mutant and is insensitive to exogenous IAA and brassinolide (BL)

**Fig. 1:**
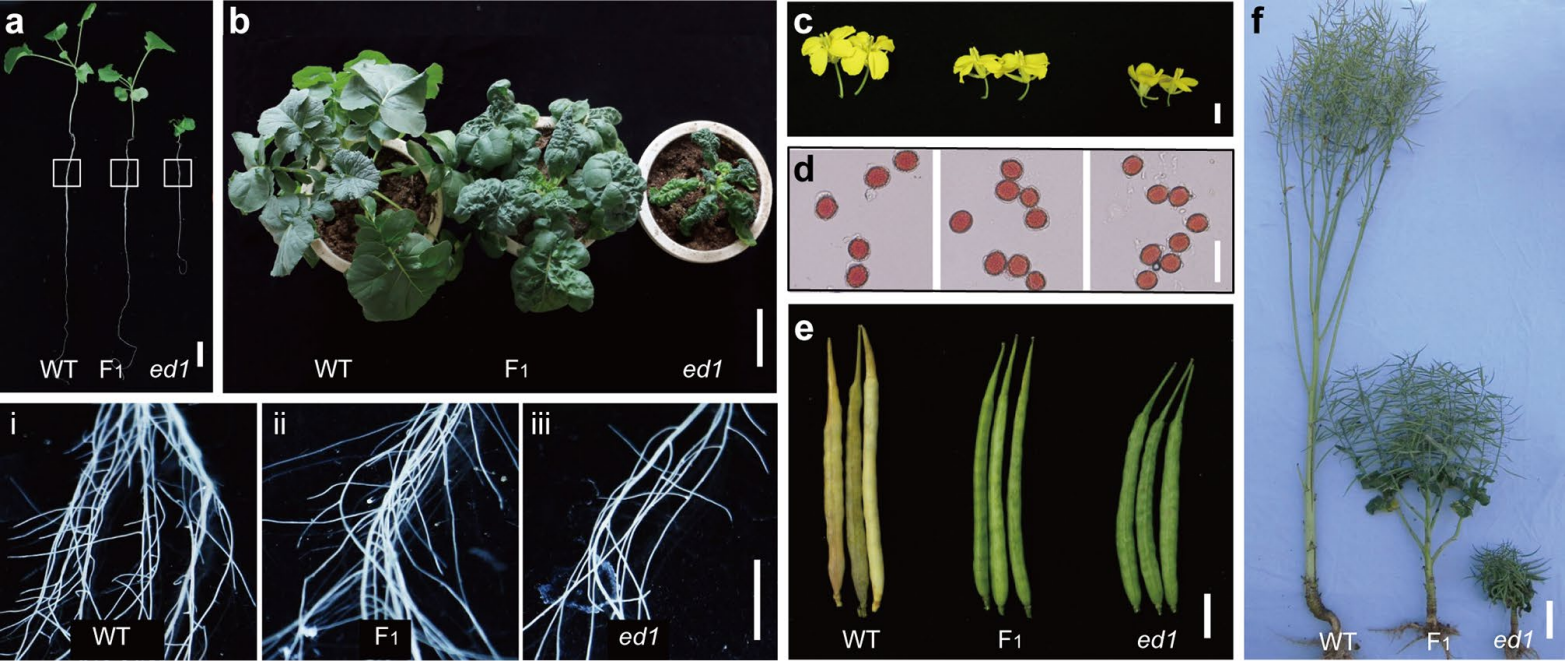
Morphological comparison of wild-type (WT), F_1_, and *ed1* plants at different developmental stages. **a** Comparison of root phenotypes of WT, F_1_, and *ed1* plants at the three-leaf stage. (**i**–**iii**) Magnified images of the framed box in **a**. **b** Comparison of leaf phenotypes and the whole-plant stature of WT, F_1_, and *ed1* plants at the vegetative stage. Comparison of flower (**c**), pollen (**d**), and silique (**e**) sizes of WT, F_1_, and *ed1* plants. **f** Comparison of WT, F_1_, and *ed1* plant heights at the mature stage. Bars = 3 cm (**a**), 10 cm (**b**, **f**), 1 cm (**c**, **e**, i–iii), and 10 μm (**d**)

We obtained the *ed1* mutant from the field and back-crossed it with the Ningyou 336 (NY336, wild-type) strain to generate a homozygous line. Compared with wild-type (WT), the *ed1* mutant showed reduced stature throughout its life cycle and displayed weaker apical dominance (Fig. 1). At the seedling stage, root elongation and lateral root outgrowth were inhibited in *ed1* (Fig. 1a, i–iii). At the vegetative stage, the most obvious phenotypes of *ed1* were the wavy and curled leaves (Fig. 1b). At flowering, the flowers and pollen grains were smaller than those of WT (Fig. 1c, d). At maturity, the *ed1* mutant displayed dwarfism, with shorter siliques (Fig. 1e, f). Briefly, all of the yield-related traits decreased (Table 1). The phenotypes of F_1_ plants were intermediate between the parental plants, indicating that the *ed1* mutation acted in a semi-dominant manner (Fig. 1; Table 1). Genetic analysis of an F_2_ population generated from a cross between WT and *ed1* showed that the curled leaf and dwarfism traits were linked and were controlled by a single semi-dominant gene (Supplementary Fig. 1).

**Table 1:** Morphological traits of WT, F_1,_ and *ed1* plants

	Plant height (cm)	Primary branch number	Secondary branch number	Number of siliques/plant	Length of silique (cm)	Number of seed/silique
WT	177.75 ± 12.58	14.00 ± 2.45	14.00 ± 5.94	530.75 ± 144.63	5.98 ± 0.13	29.45 ± 0.90
F_1_	64.60 ± 3.85	9.20 ± 1.92	4.00 ± 2.35	293.00 ± 57.44	5.94 ± 0.31	29.16 ± 1.87
*ed1*	23.60 ± 1.34**	5.40 ± 0.89**	0.00 ± 0.00**	60.60 ± 5.55**	3.93 ± 0.39**	22.88 ± 2.78**

Error bars indicate ± SD (*n* = 5). Student’s *t* test was used for statistical analysis (*P < 0.05; **P < 0.01)

The *ed1* mutant displayed curled and dark-green leaves, and had shorter hypocotyls than WT in the light (Fig. 1a, b; Supplementary Fig. 2a), suggesting that *ed1* might be defective in IAA or BR biosynthetic or signaling pathways (Reed 2001; Clouse 2011). We, therefore, treated WT and *ed1* seedlings (3 days after germination) with several concentrations of either IAA or BL added to their hydroponic culture solution for 4 days or 7 days, respectively, and measured the effect on hypocotyl lengths. The results showed that *ed1* is less sensitive to IAA or BL than WT plants (Supplementary Fig. 2b). Moreover, measurements of endogenous IAA and BR showed that *ed1* accumulates markedly higher levels of IAA than WT (Supplementary Fig. 2c). Therefore, we hypothesized that *ed1* is an IAA/BR-insensitive mutant that is involved in IAA signaling.

### Cell elongation or expansion is defective in *ed1*

**Fig. 2:**
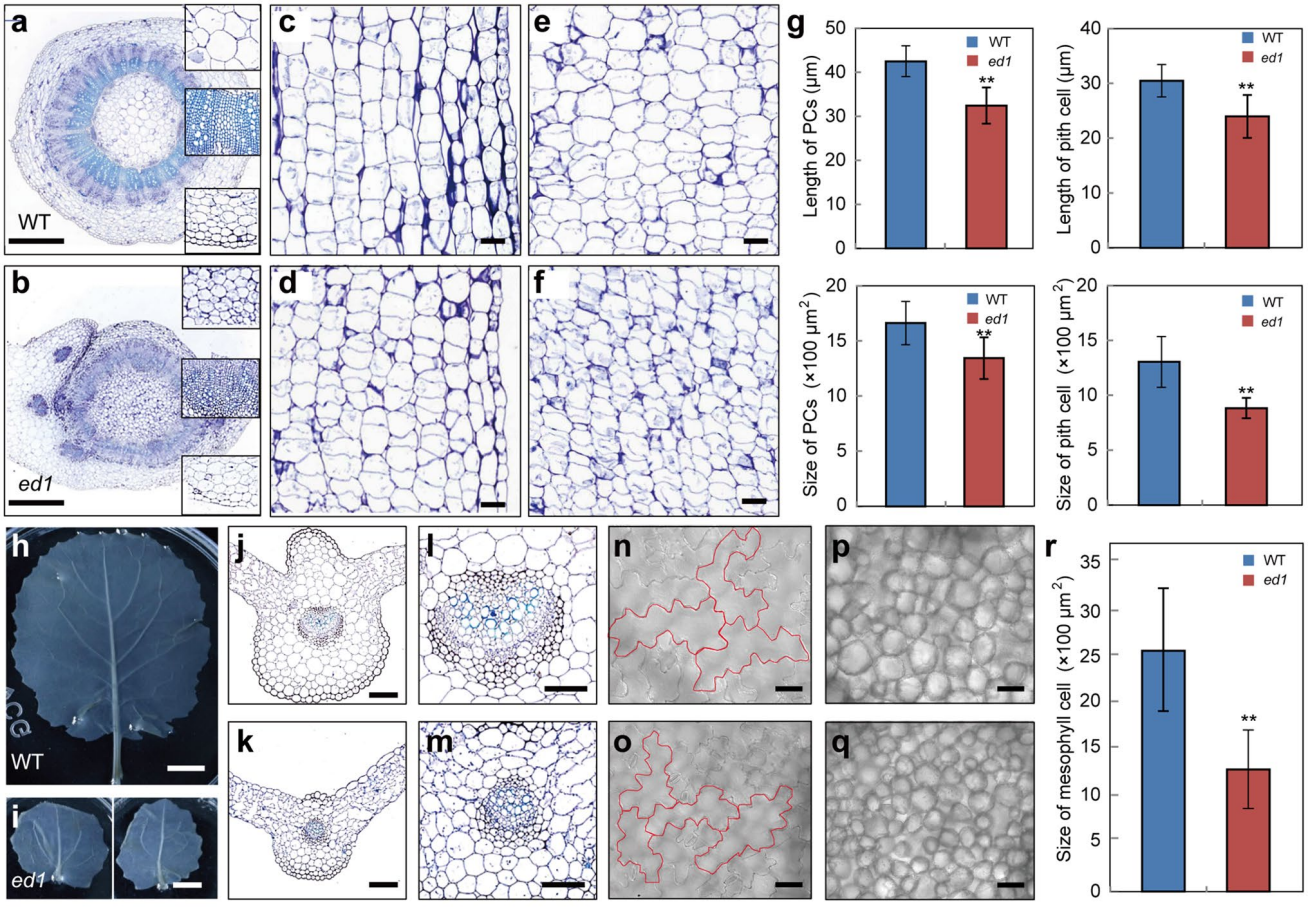
Cell elongation or expansion are defective in *ed1* plants. **a**, **b** Cross sections of WT and *ed1* stems from plants at the five-leaf stage. Framed regions from top to bottom are as follows: pith cells, xylem, and parenchymal cells. Longitudinal sections of parenchymal cells in WT (**c**) and *ed1* (**d**) stems. Longitudinal sections of pith cells in WT (**e**) and *ed1* (**f**) stems. **g** Statistical analysis of the lengths and sizes of the parenchymal and pith cells shown in **c**–**f**. Error bars ± SD (*n* = 90, 90 and 30, 56 in WT and *ed1* of PC cell length or size; *n* = 60, 159 and 56, 109 in WT and *ed1* of pith cell length or size). Student’s *t* test was used for the statistical analysis (*P ≤ 0.05; **P ≤ 0.01). **h**, **i** Whole-mount clearing of WT and *ed1* leaves (four-leaf stage). Cross sections of WT (**j**) and *ed1* (**k**) midribs. **l**, **m** Magnifications of the vascular bundles shown in **j** and **k**. Comparison of lower epidermal cells from WT (**n**) and *ed1* (**o**) leaves. Red curves indicate the contours of the lower epidermal cells. Comparison of mesophyll cells from WT (**p**) and *ed1* (**q**) leaves. **r** Statistical analysis of mesophyll cell sizes in WT and *ed1* plants. Error bars ± SD (*n* = 20). Student’s *t* test was used for the statistical analysis (*P ≤ 0.05; **P ≤ 0.01). Bars = 1 cm (**a**, **d**), 50 μm (**b**, **c**, **e**, **f**, **n**, **o**, **s**, **p**), and 200 μm (**l**, **q**, **m**, **r**) (colour figure online)

To understand the cellular basis of the *ed1* dwarfism and small leaf phenotypes, we performed a comparative histological analysis of the stems and leaves of WT and *ed1* plants at the four-leaf stage. Examination of cross sections revealed that cell radii in the *ed1* stems were significantly reduced, and differentiation of the primary phloem and xylem was repressed. In addition, the sizes of pith and parenchymal cells (PCs) decreased (Fig. 2a, b). Longitudinal sections of the stem showed that the cell lengths and sizes of the pith and PCs significantly decreased in *ed1* (Fig. 2c–g). In addition, the pith cells showed an irregular arrangement in *ed1* (Fig. 2f).

Whole-mount clearing of WT and *ed1* plants at the four-leaf stage revealed that the number of lateral veins was significantly reduced in *ed1* plants (Fig. 2h, i). Cross sections of midribs showed that their diameters decreased, but the numbers of PCs did not (Fig. 2j, k). In addition, the sizes of the vascular bundles in the midribs significantly decreased (Fig. 2l, m). Scanning the leaves showed that the sizes of the lower epidermal and mesophyll cells decreased (Fig. 2n–r).

Thus, the cells in *ed1* plants displayed a defect in cell elongation or expansion, which led to the plants’ smaller stature.

### *ED1* encodes a protein homologous to AtIAA7

**Fig. 3:**
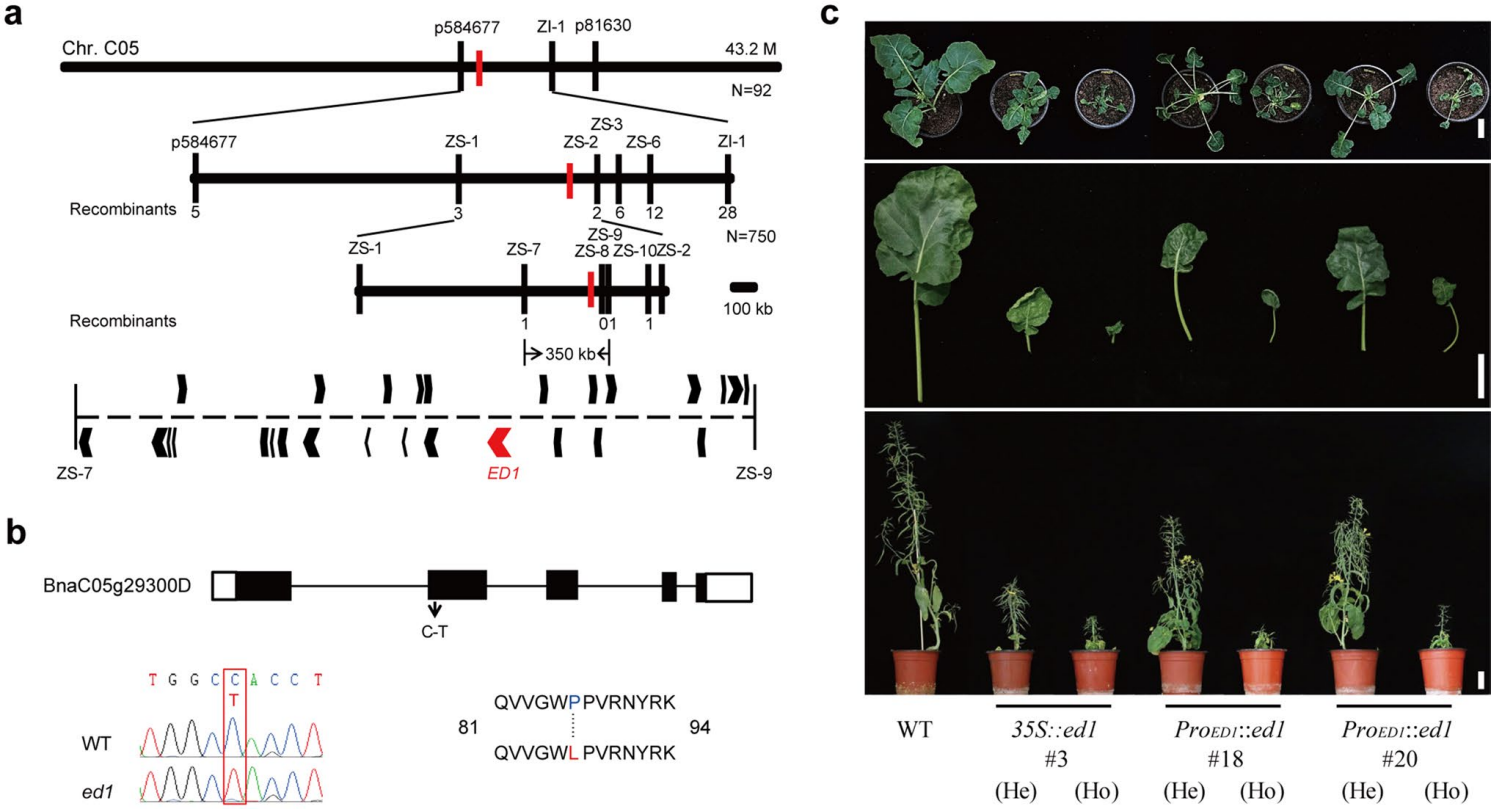
Map-based cloning and complementation tests for the *ED1* gene. **a** Fine mapping of *ED1* to a 350-kb region on chromosome C05. The region contains 27 putative open reading frames, and *ED1* is marked in red. **b** Structure of the *ED1* gene (*BnaC05g29300D*). A single-nucleotide substitution (C–T) was identified in the second exon of the *ED1* gene that converted a conserved Pro_87_ to Leu. The red box marks the changed nucleotide. **c** Morphological comparison of WT, *35S::ed1*, and *Pro*_*ED1*_*::ed1* transgenic plants. *He* heterozygous, *Ho* homozygous. Bars = 5 cm (colour figure online)

To isolate the *ED1* gene, 92 individuals from the *ed1 *× Y96 F_2_ population were genotyped using a 60-k SNP array. The major locus related to the dwarfism phenotype was located on chromosome C05 (Supplementary Fig. 3). We further narrowed the location of *ED1* to a 350-kb region between SNP markers ZS-7 and ZS-9 using 750 recessive plants (Fig. 3a; Supplementary Table 1). Twenty-seven open reading frames were predicted to be present in this region, according to the “Darmor-*bzh*” database (http://www.genoscope.cns.fr/brassicanapus/), including two *Aux/IAA* genes (*BnaC05.IAA2/BnaC05g29330D* and *BnaC05.IAA7/BnaC05g29300D*) (Supplementary Table 2). We sequenced the two candidate genes in *ed1* and found a missense mutation (C–T) in the second exon of *BnaC05.IAA7* that converted a conserved Pro (position 87) to a Leu in domain II (GW**P**PV) (Fig. 3b).

To determine whether this mutation in *BnaC05g29300D* caused the curled leaf and dwarf phenotypes, we generated transgenic plants independently expressing WT and *ed1* under the control of *CaMV35S* (*35S*) and its native promoter in Arabidopsis or *B. napus* ‘862’ (spring-type). As expected, the Arabidopsis transgenic plants that expressed *ed1* under the control of *35S* displayed the dwarf phenotype, which was a phenocopy of the *axr2/iaa7* mutant (Gray et al. 2001) (Supplementary Fig. 4a). The *ed1* transgenic lines driven by both the *35S* and the native promoters displayed curled leaves and dwarfism that phenocopied the original *ed1*, but the phenotype of the *35S*-driven transgenic line was more severe (Fig. 3c; Supplementary Fig. 4b). Therefore, we concluded that the *BnaC05.IAA7* (*BnaC05g29300D*) gene was actually *ED1* (Zhao et al. 2019).

### *ED1* acts as a repressor of IAA signaling and is degraded in response to IAA

**Fig. 4:**
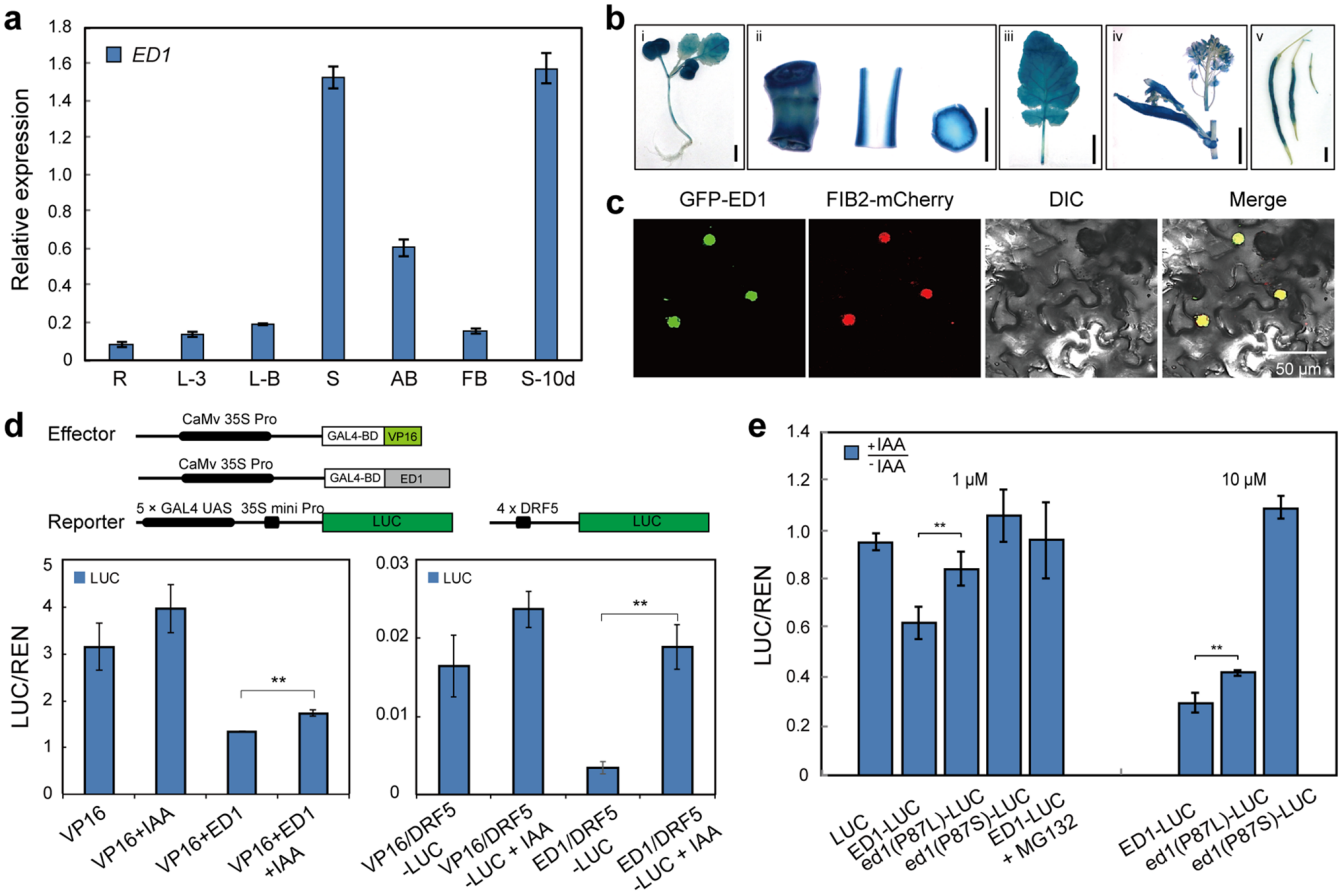
*ED1* encodes a transcriptional repressor. **a** qRT-PCR analysis showing that *ED1* was expressed at highest levels in stems. *R* roots, *L-3* leaves from three-leaf stage plants, *L-B* leaves in the bolting stage, *S* stems, *AB* axillary buds, *FB* flower buds, *S-10d* siliques 10 days after fertilization. Error bars ± SD (*n* = 3). **b** Histochemical staining of *Pro*_*ED1*_*::GUS* transgenic plants. (i) Seedling; (ii) stem; (iii) leaf; (iv) flower bud and cauline leaf; (v) siliques. Bars = 1 cm (i, ii) and 5 cm (iii, iv).** c** Subcellular localization of the GFP-ED1 fusion protein in *Nicotiana benthamiana* leaf epidermal cells. FIB2-mCherry, a nuclear marker. Bars = 50 μm. **d** Relative luciferase activity of the *Gal4*-*UAS::LUC* (left) and *DR5::LUC* (right) reporter genes in rapeseed protoplasts after IAA treatments. VP16, a transcriptional activator. Error bars ± SD (*n* = 3). Student’s *t* test was used for the statistical analysis (*P ≤ 0.05; **P ≤ 0.01). **e** Auxin-induced degradation of ED1 and ed1. Relative luciferase activity levels of ED1-LUC, ed1 (P87L)-LUC, and ed1 (P87S)-LUC fusion proteins in rapeseed protoplasts after 4 h of 1 μM (left) and 10 μM (right) IAA treatment. The ED1-LUC group was pretreated with the proteasomal inhibitor MG132 (40 μM) for 1 h before the IAA treatment. Error bars ± SD (*n* = 3). Student’s *t* test was used for the statistical analysis (*P ≤ 0.05; **P ≤ 0.01)

Quantitative RT-PCR (qRT-PCR) analyses revealed that *ED1* was expressed in various organs, including roots, leaves, axillary buds, and the reproductive organs, but at much higher levels in stems and siliques (Fig. 4a). The β-glucuronidase (*GUS*) gene driven by the *ED1* promoter was transformed into 862 (spring-type) to more precisely detect the expression levels. Consistent with the qRT-PCR results, GUS activity was detected in all organs, but predominately in the stem (Fig. 4b). Further examination of stem cross- and longitudinal sections revealed that GUS was predominately expressed in the vascular bundles (Fig. 4b, ii). In addition, high levels of GUS activity were observed in both the mesophyll and leaf veins (Fig. 4b, iii). Thus, *ED1* was constitutively expressed and might play important roles in regulating the normal growth of cells and vascular bundles.

To determine the subcellular localization of ED1, the C-terminal region of GFP was fused to the N-terminus of ED1, and the fusion gene was transiently expressed in epidermal cells of *Nicotiana benthamiana* leaves. GFP-ED1 co-localized to the nucleus with the nuclear marker FIB2-mCherry (Zheng et al. 2016) (Fig. 4c). In addition, the N-terminal region of ED1 contains an EAR motif (L × L × L), which is involved in transcriptional repression (Tiwari et al. 2004). We fused the GAL4-DNA binding domain to the N-terminus of ED1 or VP16 (a transcriptional activator) to construct effector proteins. The effector and GAL4–UAS–LUC or DR5–LUC reporter were then co-transfected into rapeseed protoplasts in the presence or absence of IAA. ED1 repressed the expression of LUC and DR5–LUC, and this repression was partially alleviated by IAA treatment, indicating that ED1 was a repressor in the auxin-signaling pathway (Fig. 4d).

We further evaluated whether ED1 and ed1 proteins could be degraded in response to auxin. We, therefore, compared the luciferase activities in rapeseed protoplasts 4 h after transfection with *ED1*–*LUC*, *ed1* (P87L)–*LUC*, and *ed1* (P87S)–*LUC* in the presence of 0, 1, or 10 µM IAA, IAA treatment induced the rapid degradation of the ED1-LUC but not the mutant ed1 (P87L)–LUC fusion proteins (Fig. 4e). This suggested that auxin rapidly destabilized the ED1 protein and that the P87L substitution in the ed1 protein affected the normal degradation rate. In addition, auxin showed no observable effects on the P87S substitution in domain II, even under high IAA concentrations (Fig. 4e).

### P-to-L substitutions in the GWPPV motifs of three BnaIAA7 homologs result in different morphologies

**Fig. 5:**
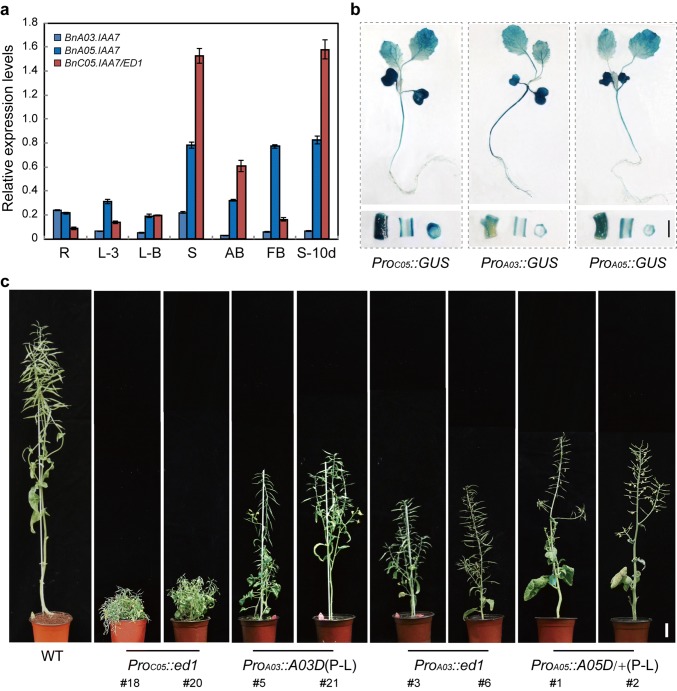
Expression pattern analyses of three *BnaIAA7*s and a morphological comparison of *Pro*_*native*_*::IAA7*s (P–L) transgenic plants. **a** qRT-PCR analyses of the expression levels of three *BnaIAA7*s in different organs. The *BnaTMA7* gene was used as an internal control. Error bars ± SD (*n* = 3). Student’s *t* test was used for the statistical analysis (*P ≤ 0.05; **P ≤ 0.01). **b** Histochemical staining of *Pro*_*C05*_*::GUS*, *Pro*_*A03*_*::GUS*, and *Pro*_*A05*_*::GUS* transgenic plants. Bar = 1 cm. **c** Different morphologies of *Pro*_*C05*_*::ed1*, *Pro*_*A03*_*::A03D* (P86L), *Pro*_*A03*_*::ed1*, and *Pro*_*A05*_*::A05D*/+ (P87L) transgenic plants. A05D/+ indicates heterozygous plants. A03, A05, and C05 indicate *BnaA03*.*IAA7*, *BnaA05*.*IAA7*, and *BnaC05*.*IAA7*, respectively. Bar = 5 cm

Genomic-synteny and BLASTP (protein–protein BLAST) analyses identified four rapeseed ED1 homologs (Supplementary Table 3; Supplementary Fig. 5). We studied whether these *BnaIAA7* homologs had similar spatio-temporal expression patterns to those of *ED1* in rapeseed by qRT-PCR analyses. These showed that two *BnaIAA7* homologs (C01 and C07) were barely expressed in the tested tissues (data not shown), while *BnaA03.IAA7* had higher expression levels in roots and stems than in other tissues, *BnaC05.IAA7* showed significant expression in stems and siliques, and *BnaA05.IAA7* was predominately expressed in stems, flower buds, and siliques (Fig. 5a). This indicated that the expression of these homologs varied greatly between tissues. To analyze the expression of each BnaIAA7 in more detail, we constructed *Pro*_*BnaIAA7s*_*::GUS* plasmids and transformed them into ‘862’. Histochemical GUS staining of the seedlings showed high expression of *BnaA03.IAA7* in young roots, whereas expression of the other homologs was low (Fig. 5b). At the bolting stage, strong GUS signals were detected in the stem of the *Pro*_C05_::*GUS* line, but other lines showed weaker signals (Fig. 5b). Thus, the expression analyses and histochemical GUS staining indicated that the three *BnaIAA7* homologs might have diverse functions in rapeseed.

To confirm the defective phenotypes of other two BnaIAA7 copies, we next created variants of the BnaIAA7s that mimicked the *ed1* mutation by replacing P with L in the GW**P**PV motif and expressed them in rapeseed using native promoters. In addition, we constructed a plasmid that expressed mutant *ed1* controlled by the *BnaA03*.*IAA7* promoter. We then compared the architecture of the homozygous transgenic plants with WT. *Pro*_*C05*_*::ed1* displayed the shortest stems, ~ 16.5% of the WT stem length, while both *Pro*_*A03*_*::A03D* (P86L) and *Pro*_*A03*_*::ed1* were semi-dwarf compared with WT (Fig. 5c). Thus, the different expression levels of *BnaC05.IAA7* and *BnaA03*.*IAA7* in stems might be responsible for the distinct differences in PH (Supplementary Fig. 6). Notably, all eight of the *Pro*_*A05*_*::A05D* (P87L) T_0_ positive lines displayed sterility and had decreased PH. This was consistent with the expression analyses, showing that *BnaA05.IAA7* was most highly expressed in floral buds (Fig. 5a).

We, therefore, hypothesized that the three homologs of *BnaIAA7* might have redundant and divergent functions in regulating rapeseed PH, where *BnaC05.IAA7* predominately regulates stem elongation.

### IAA affects interactions between BnaIAA7s and BnaTIR1/AFBs

**Fig. 6:**
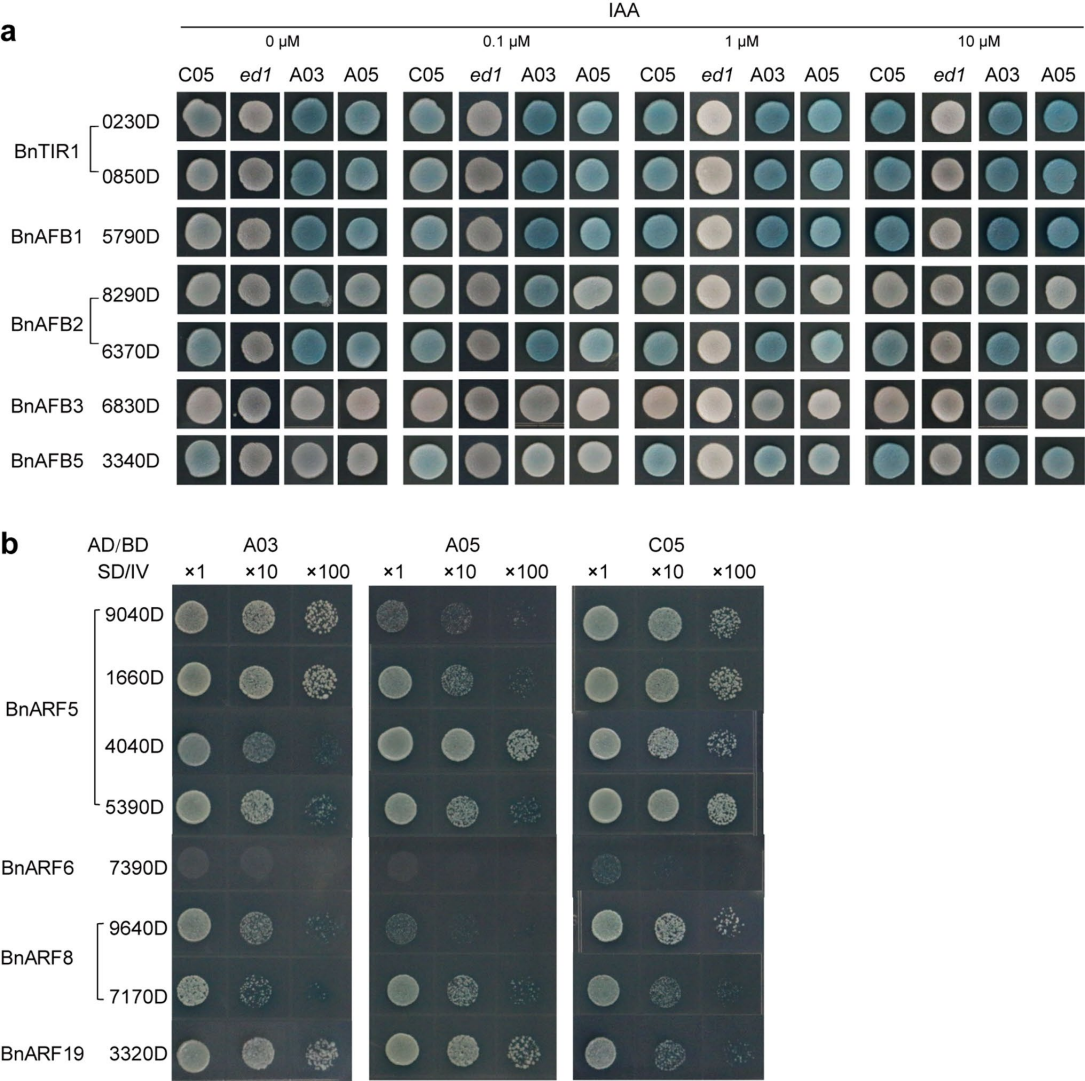
Interactions between BnaIAA7s and BnaTIR1/AFBs or BnaARFs in yeast. **a** Differences in BnaIAAs–BnaTIR1/AFBs interactions. Selective media (SD/Gal/Raff/–Ura–His–Trp + X-Gal + BU salts) containing increasing concentrations of IAA. The abbreviations 0230D, 0850D, 5790D, 8290D, 6370D, 6830D, and 3340D indicate *BnaA04g00230D*, *BnaCnng60850D*, *BnaA03g25790D*, *BnaA02g28290D*, *BnaC02g36370D*, *BnaA09g46830D*, and *BnaA03g23340D*, respectively. **b** Differences in BnaIAAs–BnaARFs interactions. SD IV, selective medium (SD/−Trp–Leu–His–Ade + x-α-gal). The abbreviations 9040D, 1660D, 4040D, 5390D, 7390D, 9640D, 7170D, and 3320D indicate *BnaC08g19040D*, *BnaA07g11660D*, *BnaA06g14040D*, *BnaC05g15390D*, *BnaA08g17390D*, *BnaC03g59640D*, *BnaC06g27170D*, and *BnaA06g13320D*, respectively. A03, A05, and C05 indicate *BnaA03*.*IAA7*, *BnaA05*.*IAA7*, and *BnaC05*.*IAA7*, respectively

The TIR1/AFB family members function as auxin receptors (Dharmasiri et al. 2005). We, therefore, used yeast two-hybrid (Y2H) assays to analyze interactions between various BnaTIR1/AFB-BnaIAA7 pairs. After eliminating the pairs that showed no interactions, we tested the interactions between seven BnaTIR1/AFBs and the BnaIAA7s across an auxin concentration gradient. Different dose responses were observed for each pair. In the absence of auxin, BnaA03.IAA7 and, to a lesser extent, BnaA05.IAA7, interacted strongly with BnaTIR1, BnaAFB1, and BnaAFB2. In contrast, BnaC05.IAA7 showed a slightly stronger interaction with BnaAFB5 in the absence of auxin (Fig. 6a). As the IAA concentration increased, the strength of the interactions between the BnaIAA7s and most BnaTIR1/AFBs increased, with the exception of BnaAFB2 (*BnaA02g28290D*) which interacted increasingly weakly with all three BnaIAA7s with increasing auxin concentration (Fig. 6a). In contrast, the interaction of BnaAFB2 (*BnaC02g36370D*) with the BnaIAA7 homologs did not significantly vary with auxin concentration (Supplementary Fig. 7).

The interactions between the BnaIAA7s and both BnaTIR1 and BnaAFB1 were particularly strong, and BnaC05.IAA7 showed the most obvious increase in interaction with auxin concentration (Fig. 6a; Supplementary Fig. 7). Conversely, BnaAFB3 only interacted with the BnaIAA7s at high auxin levels. Moreover, the mutant ed1 did not interact with any BnaTIR1/AFB1 (Fig. 6a; Supplementary Fig. 7). Thus, each BnaIAA7 homolog appeared to interact differently with the auxin receptors, and the mutation in ED1 domain II might have abolished the interactions between this domain and auxin receptors in yeast.

### The three BnaIAA7s interact in different ways with BnaARFs

The Aux/IAAs regulate auxin-response genes by repressing the ARFs (Weijers et al. 2005). In this study, we aimed to determine which BnaARFs interacted with the BnaIAA7s using Y2H assays. We chose products of *BnaARF5*, *BnaARF6*, *BnaARF8*, *BnaARF18*, and *BnaARF19* as the candidate interactors, because these genes are involved in cell elongation or vascular development (Nagpal et al. 2005; Wilmoth et al. 2005; Donner et al. 2009), which are related to the phenotypes of the *ed1* plants. All of the tested BnaARFs, except for BnaARF18, exhibited interaction with the BnaIAA7s or ed1 in yeast (Supplementary Fig. 8b). After 4 days on selective plates, BnaA03.IAA7 interacted strongly with BnaARF7s (*BnaC08g19040D* and *BnaA07g11660D*), as compared with BnaA05.IAA7 and BnaC05.IAA7 (Fig. 6b). BnaA05.IAA7 had the strongest interaction with BnaARF5 (*BnaA06g14040D*), while BnaC05.IAA7 showed the strongest interaction with BnaARF5 (*BnaC05g15390D*) (Fig. 6b). In addition, BnaC05.IAA7 weakly interacted with BnaARF6 (*BnaA08g17390D*), whereas the other two BnaIAA7s did not. Furthermore, BnaC05.IAA7 interacted more weakly with BnaARF8 (*BnaC06g27170D*) and BnaARF19 (*BnaA06g13320D*) than BnaA03.IAA7 and BnaA05.IAA7, but more strongly than the other two BnaIAA7s with BnaARF8 (*BnaC03g59640D*) (Fig. 6b).

### BnaIAA7s affect BR signaling via the interaction between BnaARF8 and BnaBZR1

**Fig. 7:**
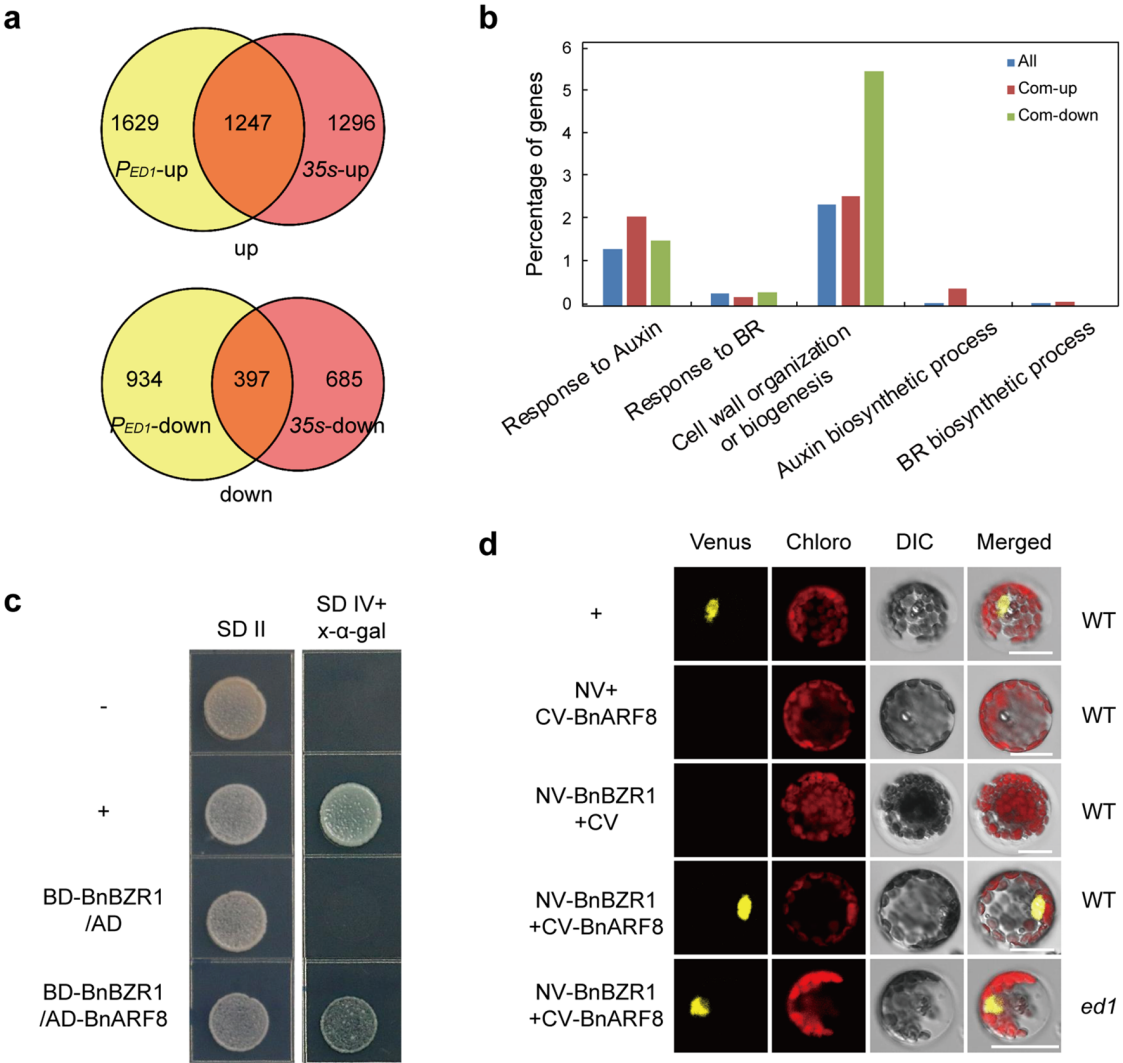
Differentially expressed genes related to Aux- or BR responses and interaction between BnaARF8 and BnaBZR1. **a** Venn diagrams showing the genes that were differentially expressed in “862” vs. *Pro*_*ED1*_*::ed1* or *35S::ed1*. Up, genes whose expression was upregulated in *Pro*_*ED1*_*::ed1* and *35S::ed1* plants; down, genes whose expression was downregulated in *Pro*_*ED1*_*::ed1* and *35S::ed1* plants. **b** Gene ontology analysis of the 1644 genes that were differentially expressed in both *Pro*_*ED1*_*::ed1* and *35S::ed1*. **c** Yeast two-hybrid assay, showing that BnaARF8 (*BnaC03g59640D*) interacts with BnaBZR1 (*BnaAnng32010D*). *SD II* control medium (SD/−Trp–Leu), *SD IV* selective medium (SD/−Trp–Leu–His–Ade). **d** Bimolecular fluorescence complementation (BiFC) assays showing that BnaARF8 can interact with BnaBZR1 in the nuclei of rapeseed WT or *ed1* protoplasts. −, Negative control; +, positive control

The *ed1* mutant had an abnormal plant architecture that was similar to BR-related phenotypes, including short stems and curled and dark-green leaves. We, therefore, performed an RNA-Seq analysis in which WT was compared separately with *Pro*_*ED1*_::*ed1* and *35S::ed1* homozygous plants to determine whether the *ed1* mutation affected the BR-response pathway. The transcriptome analysis identified 397 genes that had decreased expression levels (> 1.5-fold) compared to wild type in both *Pro*_*ED1*_::*ed1* and *35S::ed1* and 1247 genes that had increased expression (> 1.5-fold) relative to wild type in both transgenic lines (Fig. 7a; Supplemental Datasheet). A gene ontology (GO) analysis showed that some of these genes responded to auxin or BR, or were involved in hormone biogenesis, while other genes involved in cell wall organization were highly enriched (Fig. 7b). In addition to the differentially expressed genes that were related to auxin or BR biosynthesis and signaling, we identified differentially expressed genes related to cell elongation/expansion that had previously been reported to be downstream targets of BZR1 (Sun et al. 2010) (Table 2; Supplementary Table 4).

**Table 2:** Auxin or BR-biosynthesis and signaling genes were aberrantly expressed in Pro_*ED1*_::*ed1* or 35S::*ed1* plants

35S::*ed1*/P_*ED1*_::*ed1*	IAA		BR		BZR1/BES1 targets		
	Biosynthesis	Signaling	Biosynthesis	Signaling	Cell elongation	Cell biosynthesis/expansion	Development
UP	YUC6, YUC8, YUC9, TAA1, CYP79B2, CYP79B3, SOT16, AO1, AAO2	BRu6, GH3, IAA11, IAA19, IAA29	CYD709B2, DWF4	BEH3	IBH1, BEE2	–	–
Down	CYP71A13	SAUR-like	SWF1	BSK2	PRE5, AIF1	EXPA5, EXPA11, EXP3, XTH9, XTH32, HEXO3, PME2	MIF1

Notably, no change in the expression level of either *BnaBZR1* or *BnaBES1* was detected, and expression levels of *BnaARF*s were unchanged in the two transgenic lines. The BnaARFs are the downstream targets of BnaIAAs, and we hypothesized that some BnaARFs might interact with BnaBZR1 in rapeseed. Y2H assays revealed that BnaARF8 directly interacted with BnaBZR1 (Fig. 7c). Similarly, BiFC assays showed that BnaARF8 interacted with BnaBZR1 in the nuclei of rapeseed protoplasts, and even in *ed1* protoplasts, indicating that the domain II of ED1/BnaC05.IAA7 does not mediate these interactions (Fig. 7d). These results indicated that the interaction between BnaARF8 and BnaBZR1 might act as a bridge between auxin and BR signaling in rapeseed growth and development.

## Discussion

In this study, we identified an *ed1* mutant in rapeseed that displayed extreme dwarfism, a curled leaf phenotype and short hypocotyls. These phenotypes were similar to those of some Arabidopsis mutants deficient in auxin/BR biosynthesis or signaling (Clouse 2011; Reed 2001). Exogenous hormonal treatments showed that, compared with WT, hypocotyl elongation of the *ed1* mutant was insensitive to IAA and BL. This observation was consistent with previous studies of the Arabidopsis *iaa7*/*axr2* mutant (Nemhauser et al. 2004; Nakamura et al. 2006). Significantly, the changes caused by the two treatments on *ed1* plants were the same (Supplementary Fig. 2b). In addition, the RNA-Seq analysis revealed that several genes related to IAA and BR synthesis or signaling were aberrantly expressed in *Pro*_*ED1*_::*ed1* and *35S::ed1* plants (Table 2). The finding that the expression of IAA biosynthesis genes increased implies that there is a negative feedback loop controlling auxin levels in the *ed1* mutant. Thus, the IAA- and BR-signaling pathways may be simultaneously deficient in the *ed1* mutant, and auxin might mediate growth responses through BR signaling or the two hormones may have overlapping target genes that regulate cell elongation or expansion.

Our study showed that *ed1*, which has a mutation in domain II (GW**P**PV motif), is a gain-of-function mutant. In Arabidopsis, most Aux/IAAs repressor mutants have a mutation in either of the two Pro residues in domain II, which causes them to exhibit severe phenotypes (Reed 2001). Domain II is a degron motif that directly contacts TIR1 and auxin; therefore, a mutation in domain II can abolish the interaction between Aux/IAA repressors and TIR1/AFBs (Tan et al. 2007; Calderon Villalobos et al. 2012). Our interaction assays showed that the mutant ed1 protein lost its ability to bind BnaTIR1/AFBs, but could still interact with BnaARFs (Fig. 6; Supplementary Fig. 8b). Therefore, the mutation in ed1 probably abolished the interaction with the SCF^TIR1/AFBs (auxin)^ complexes in yeast. Conversely, degradation assays in rapeseed protoplasts revealed that the P87L substitution in domain II decreased the degradation rate compared with WT, and the protein with the P87S substitution could not be degraded after IAA treatment. Thus, variation at residue 87 might result in different degradation rates. Therefore, the mutant ed1 (P87L) might have a weak interaction with the SCF^TIR1/AFBs (auxin)^ complexes, at least in rapeseed (Fig. 4e).

Auxin and BR interact in some plant growth and developmental process, including hypocotyl and root elongation in Arabidopsis and leaf angle regulation in rice (Vert et al. 2008; Oh et al. 2014; Zhang et al. 2015). In Arabidopsis, the *bzr1*-*1D* mutant exhibits reduced PH, and *bes1* and *bzr1* mutants have defects in phloem and xylem differentiation (Saito et al. 2018). Transcriptome analyses revealed that the expression levels of *BZR1* downstream targets involved in cell elongation or expansion decreased in the *axr2/iaa7* mutant and *Pro*_*ED1*_*::ed1* plants (Table 2; Supplementary Table 5). In addition, the *arf6*-*2* or *arf6*-*2 arf8*-*3* mutants display decreased internodes or infertile flowers (Nagpal et al. 2005). In our study, BnaARF8 directly interacted with BnaIAA7s and BnaBZR1. Notably, the differentiation of the primary phloem and xylem was arrested, and the vascular bundle size was reduced in *ed1* mutant leaves (Fig. 2j–m). A recent study revealed that the mutation of the degron motif of PtoIAA9 significantly repressed secondary xylem development in poplar via interaction with PtoARF5s (Xu et al. 2018). ARF5 could recruit BES1 to its target promoter when treated with IAA or BR in Arabidopsis (Walcher and Nemhauser 2012). Our study also found that three BnaIAA7s interact with BnaARF5s to different extents (Fig. 6b). Therefore, the BnaIAA7 proteins might be involved in BR-mediated growth responses via BnaBZR1 in rapeseed. In Arabidopsis, the *bzr1* and *bes1* single- or double-deficient mutants did not display obvious phenotypes in plant architecture, which may be the result of the presence of four additional *BES*-*like* homologs in the Arabidopsis genome (Saito et al. 2018). Similarly, both *BnaARF*s and *BnaBZR*s have many homologs in the rapeseed genome making it difficult to obtain mutants with obvious phenotypes. Intriguingly, *bes1*-RNAi lines that show decreased expression of several BZR/BES-like genes exhibit defects in stem elongation, indicating that simultaneously knocking down these functional redundant genes will obtain expected phenotypes (Wang et al. 2013). In the future, loss-of-function mutants of *BnaBZR1*/*BES1*-like homologs will be created by CRISPR/Cas9-mediated mutagenesis for genetic analysis between *bnabzr/bes1* and *bnaiaa7*s to illuminate the roles of BZR1/BES1 genes in vascular development.

Most duplicated genes diverge in their expression levels (Blanc and Wolfe 2004; Zheng et al. 2016). Our expression pattern analyses showed that the expression of the three BnaIAA7 homologs varied between organs (Fig. 5a). Promoter sequence comparisons also showed that there were major differences between the three *BnaIAA7*s (Supplementary Fig. 9). Variants with substitutions in domain II revealed that *ED1/BnaC05.IAA7* was a major factor in stem and silique elongation during rapeseed growth and development, while *BnaA05.IAA7* was essential for rapeseed reproduction. In Arabidopsis, the overexpression of a poplar IAA14 (PtrIAA14) which had many amino acid differences with AtIAA14 resulted in reduced fertility (Liu et al. 2015). Acetic acid magenta staining assays revealed that the pollen of *Pro*_*A05*_::*A05D* (P87L) transgenic plants was normal (data not shown); thus, the BnaA05.IAA7 mutation might affect the embryo sac. In addition, there was only a slight difference between *Pro*_*A03*_::*ed1* and *Pro*_*A03*_::*A03D* (P86L) plants in PH, but an obvious difference in leaf morphology (Fig. 5c; Supplementary Fig. 10), suggesting that ED1 and BnaA03.IAA7 had functional differences. The interaction assays in yeast also showed that the three BnaIAA7s interacted differently with BnaARF5/19, whose Arabidopsis homologs play roles in regulating vascular development and leaf expansion (Wilmoth et al. 2005; Donner et al. 2009). Meanwhile, the three BnaIAA7 proteins also had several amino acid differences (Supplementary Fig. 5), indicating that the BnaIAA7s had functional differences in repressing BnaARF activity levels (Muto et al. 2007).

Recently, a G-to-E mutation in the GWPPV motif of BnaA3.IAA7 was found to contribute to yield heterosis by improving plant architecture with decreased PH and branch angle (Li et al. 2018). Notably, in our study, the *Pro*_*A03*_*::A03D* (P86L) homozygous plants exhibited a semi-dwarf phenotype with major changes in leaf morphology but no change in branch angle (Supplementary Fig. 10), The phenotypic differences between the two mutant forms in the BnaA03.IAA7 degron motif might be due to the protein conformation changes resulting from the different properties of these residues, as Gly (G) affects the flexibility of Aux/IAAs, while the first Pro (P) is involved in packing auxin into the TIR1 pocket (Tan et al. 2007). Significantly, the semi-dominant *BnaA03.IAA7* (P86L) mutation also has the potential application to breed decreased PH in rapeseed, and further studies will be performed to detect whether it can increase the yield.

### Author contribution statement

MZ, MH, JZ, HP, and WH designed the experiments; MZ, MH, HY, MT, LZ, XL, and JL performed the experiments; HL analyzed the RNA-Seq data; XS and SF characterized the agronomic traits; MZ and MH wrote the manuscript, and WT revised the manuscript. All authors read and approved the final manuscript.

## Electronic supplementary material

Below is the link to the electronic supplementary material.

Supplementary material 1 (DOCX 11722 kb)

Supplementary material 2 (XLSX 68 kb)

Supplementary material 3 (XLSX 2513 kb)
