# Supplementary material for: Three BnaIAA7 homologs are involved in auxin/brassinosteroid-mediated plant morphogenesis in rapeseed (Brassica napus L.)
Source: Plant Cell Rep. 2019 Apr 22;38(8):883–97. doi: 10.1007/s00299-019-02410-4 (PMC6647246; doi:10.1007/s00299-019-02410-4)
Supplement: Supplementary file 1 — Supplementary material 1 (DOCX 11722 kb) [file 299_2019_2410_MOESM1_ESM.docx]

**Supplementary Material**

**Three *BnaIAA7* homologs are involved in auxin/brassinosteroid-mediated plant morphogenesis in rapeseed (*Brassica napus L.*)**

Ming Zheng^1, #^, Maolong Hu^2, #^, Hongli Yang^1^, Min Tang^1^, Liang Zhang^1^, Hongfang Liu^1^, Xiaokang Li^1^, Jinglin Liu^1^, Xingchao Sun^1^, Shihang Fan^1^, Jiefu Zhang^2^, William Terzaghi^3^, Huiming Pu^2,*^ and Wei Hua^1,*^


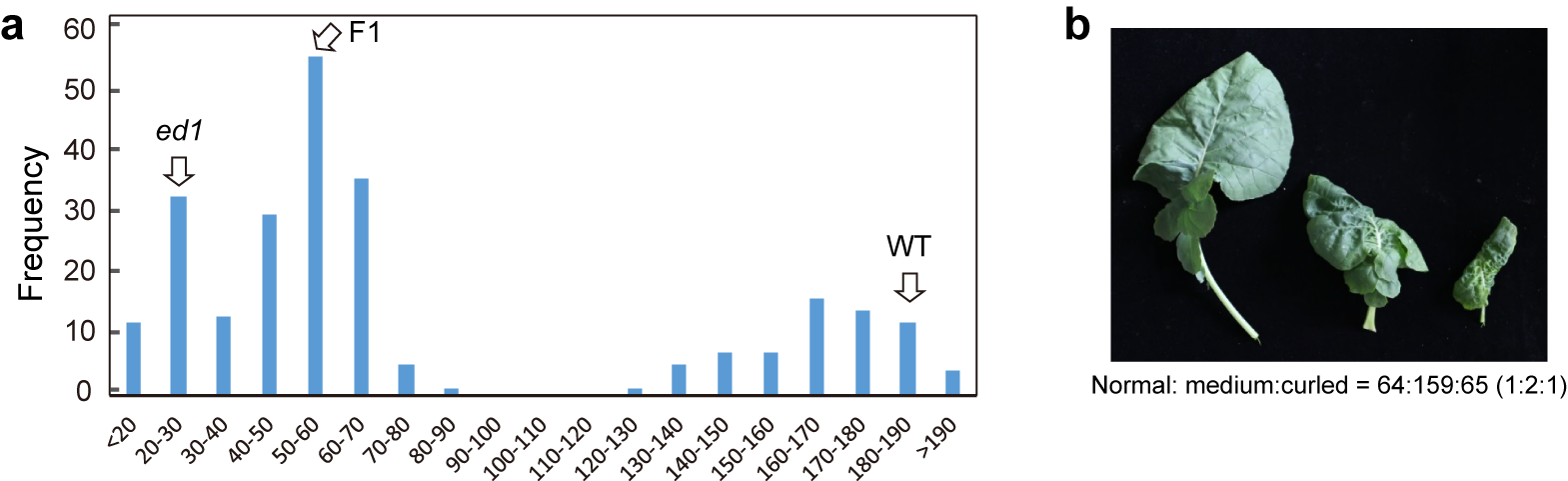
**Supplemental Figure 1.** **Morphological comparison of wild-type (WT), F_1_, and *ed1* plants and genetic analysis of wild type (WT) and the *ed1* mutant.**

a, Frequency distribution of plant heights in the F_2_ population (*ed1*× WT).

b, Segregation of leaves in the F_2_ population (*ed1*× WT).


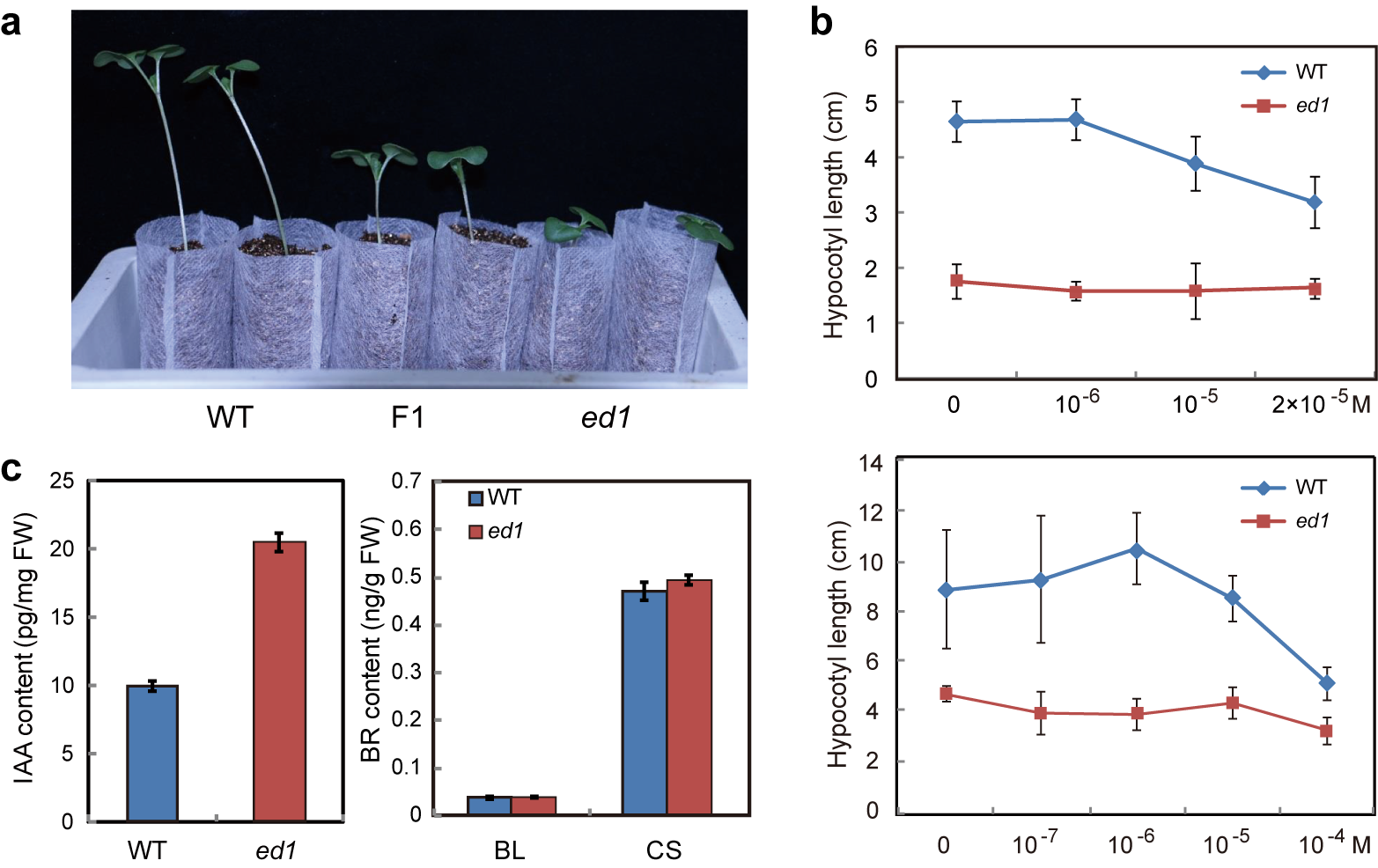


**Supplemental Figure 2. Hormonal analyses and responses of WT and *ed1* plants.**

a Phenotypes of WT, F_1_ and *ed1* hypocotyls under light.

b WT and *ed1* seedlings were grown under light for 8 d in plant nutrient solution, with the experimental groups being treated independently with 1, 10, and 20 nM IAA for 4 d (Top). WT and *ed1* seedlings were grown in the dark for 7 d in plant nutrient solution, with the experimental groups being treated independently with 0, 0.1, 10, 100, and 10,000 nM BL (Bottom). Error bars ± SD (n = 3).

c Endogenous free-IAA and BR (BL and CS) concentrations in leaf tips of WT and *ed1* at the 5-leaf stage using liquid chromatography-mass spectrometry (LC-MS). FW, fresh weight. Error bars ± SD (n = 3).


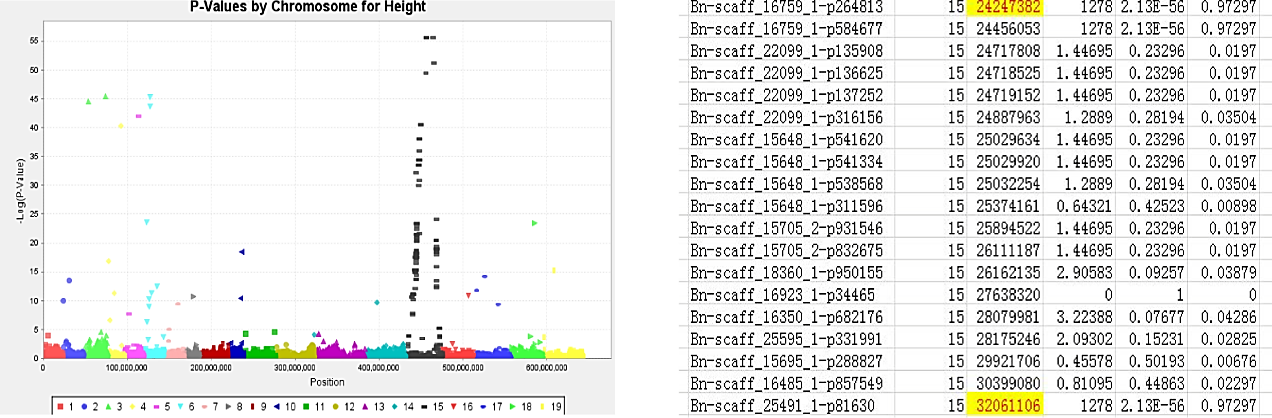


**Supplemental Figure 3. Linkage analysis of the dwarf phenotype using a 60 k SNP array.**

Yellow shading indicates the linkage probes on Chr. C05 (right).


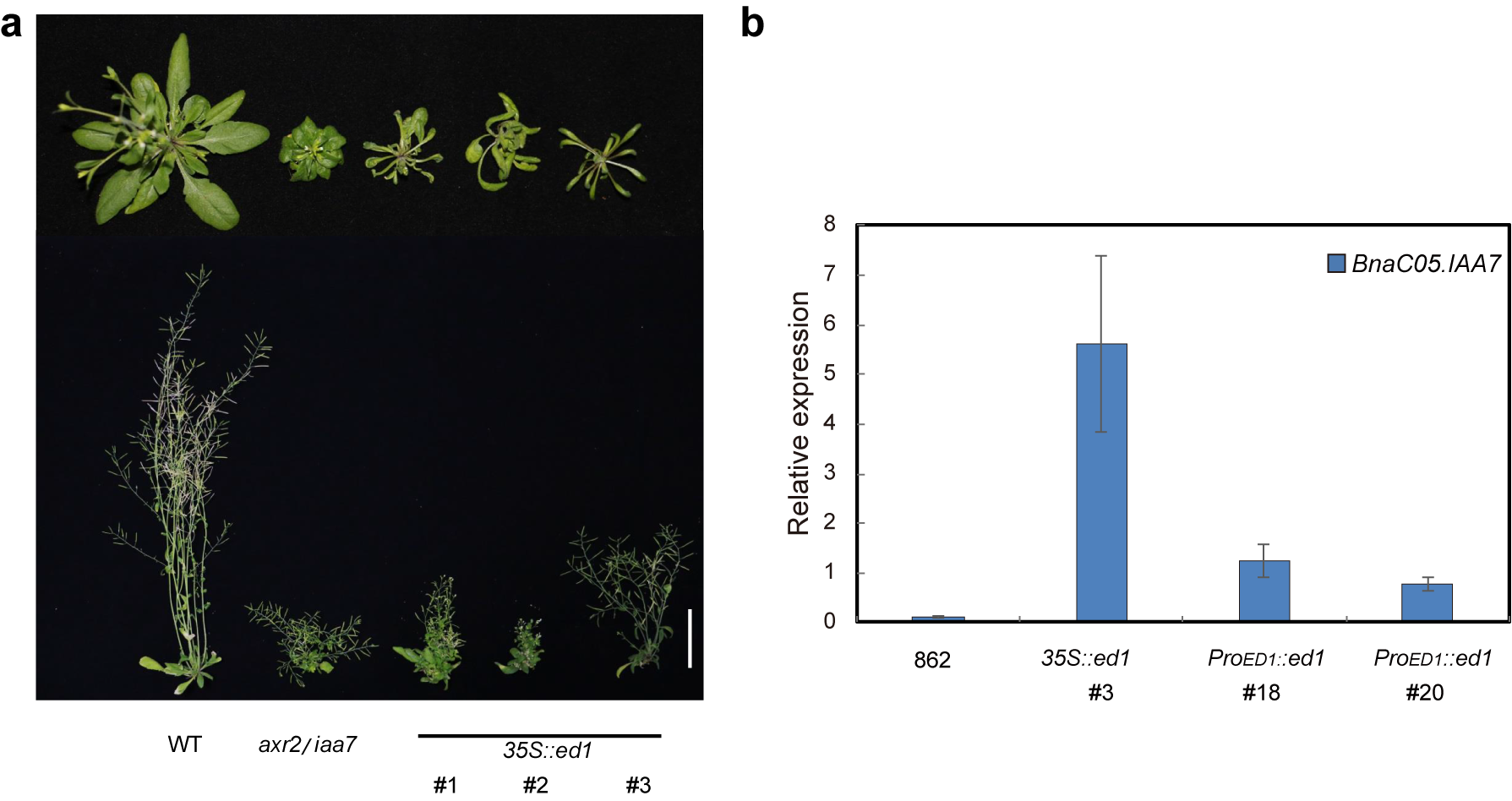


**Supplemental Figure 4. Phenotypes of 3*5S::ed1* homozygous transgenic plants in Arabidopsis (a) and qPCR analysis of *ed1* in rapeseed transgenic plants (b).**

Error bars ± SD (n = 3).


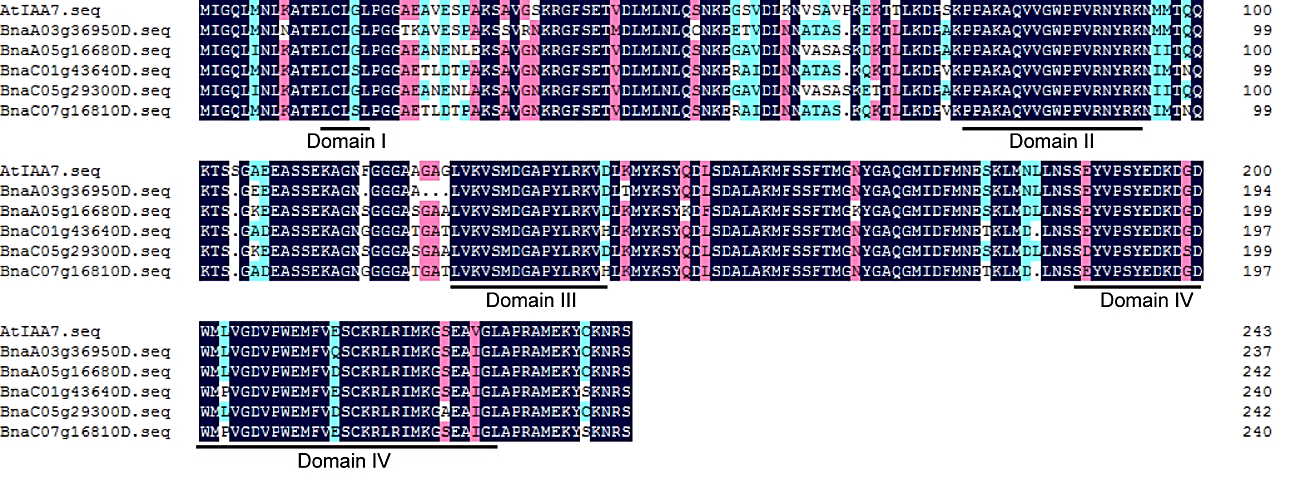
**Supplemental Figure** 5. **Alignment of AtIAA7 and BnaIAA7s amino acid sequences.**

The solid underline bars mark the four conserved domains.


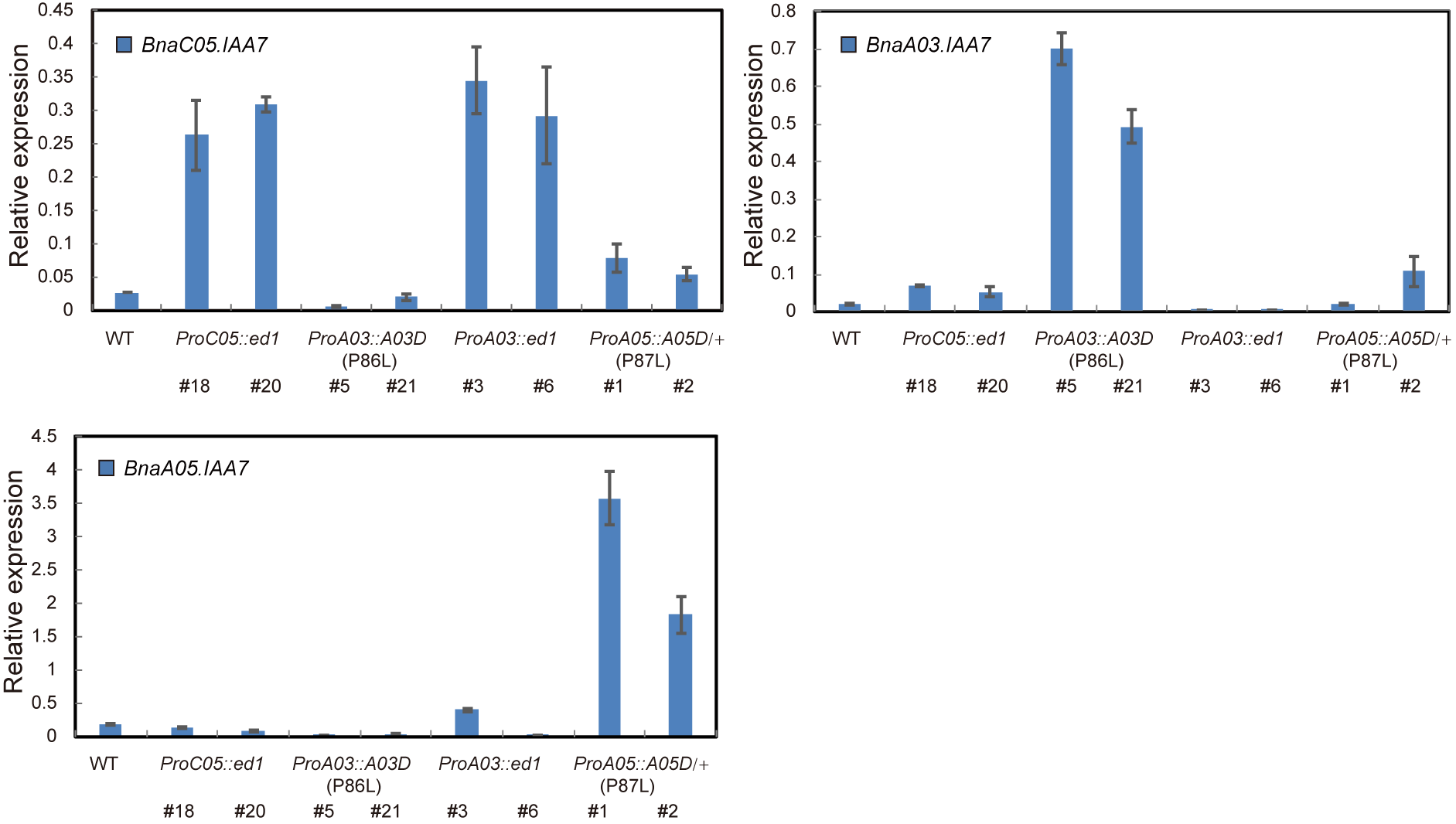
**Supplemental Figure** 6. **qPCR analysis of *BnaIAA7s* in rapeseed transgenic plants.**

A03, A05, and C05 indicate *BnaA03.IAA7*, *BnaA05.IAA7*, and *BnaC05.IAA7*, respectively. Error bars ± SD (n = 3).


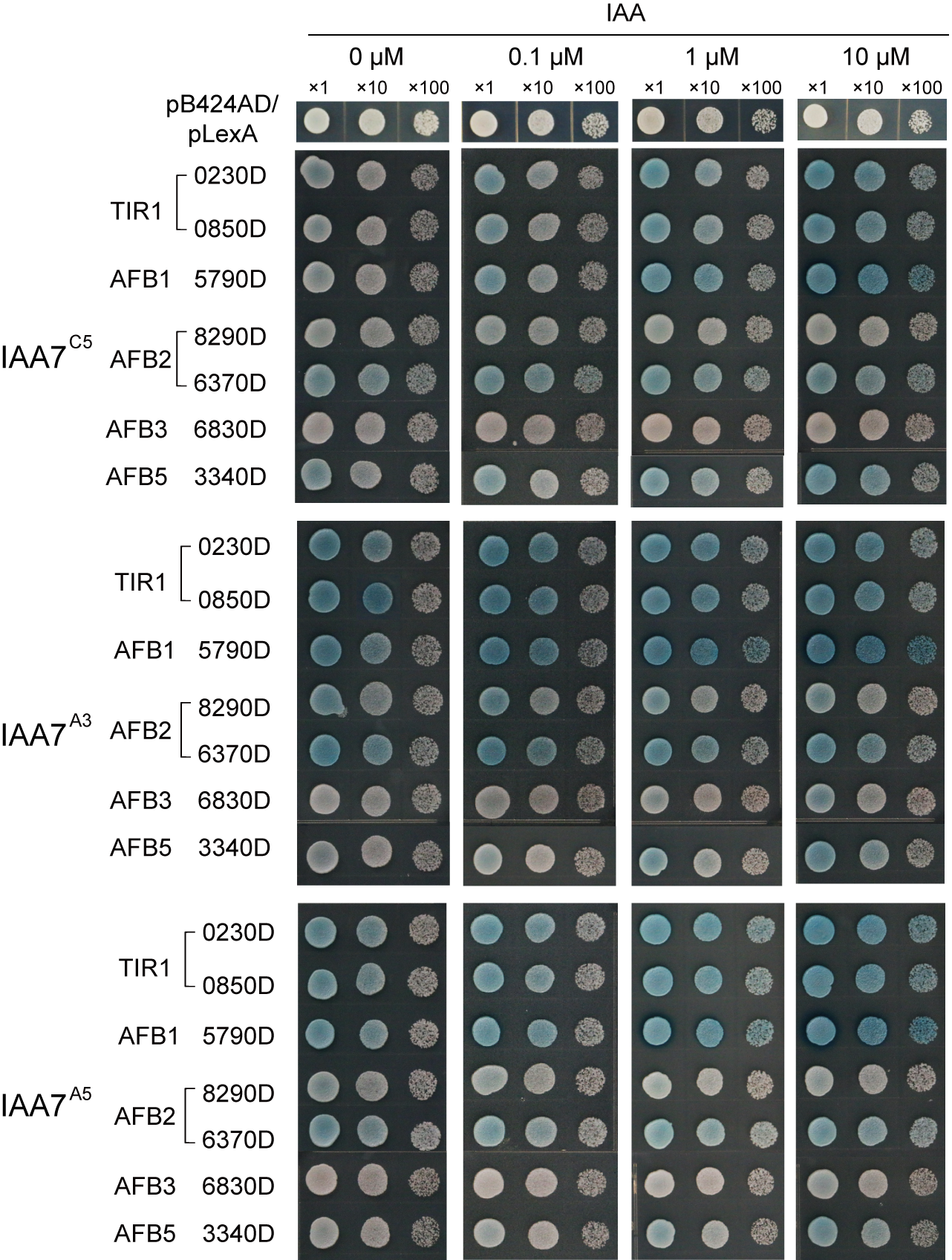


**Supplemental Figure 7. Differences in BnaIAAs-BnaTIR1/AFBs interactions.**

Selective media (SD/Gal/Raff/-Ura-His-Trp + X-Gal + BU salts) contained increasing concentrations of IAA. 0230D, 0850D, 5790D, 8290D, 6370D, 6830D and 3340D indicated *BnaA04g00230D, BnaCnng60850D, BnaA03g25790D, BnaA02g28290D, BnaC02g36370D, BnaA09g46830D* and *BnaA03g23340D*, respectively. A03, A05 and C05 indicated *BnA03.IAA7*, *BnA05.IAA7* and *BnC05.IAA7*, respectively.


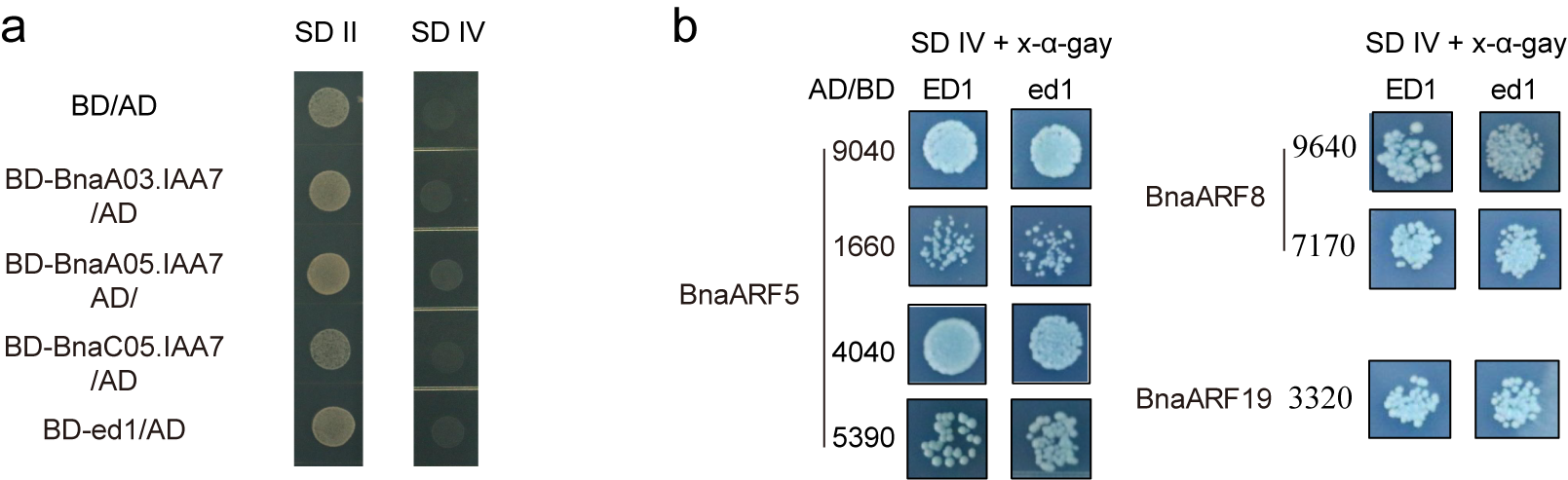


**Supplemental Figure 8.** Interactions between ED1/ed1 and BnaARFs.


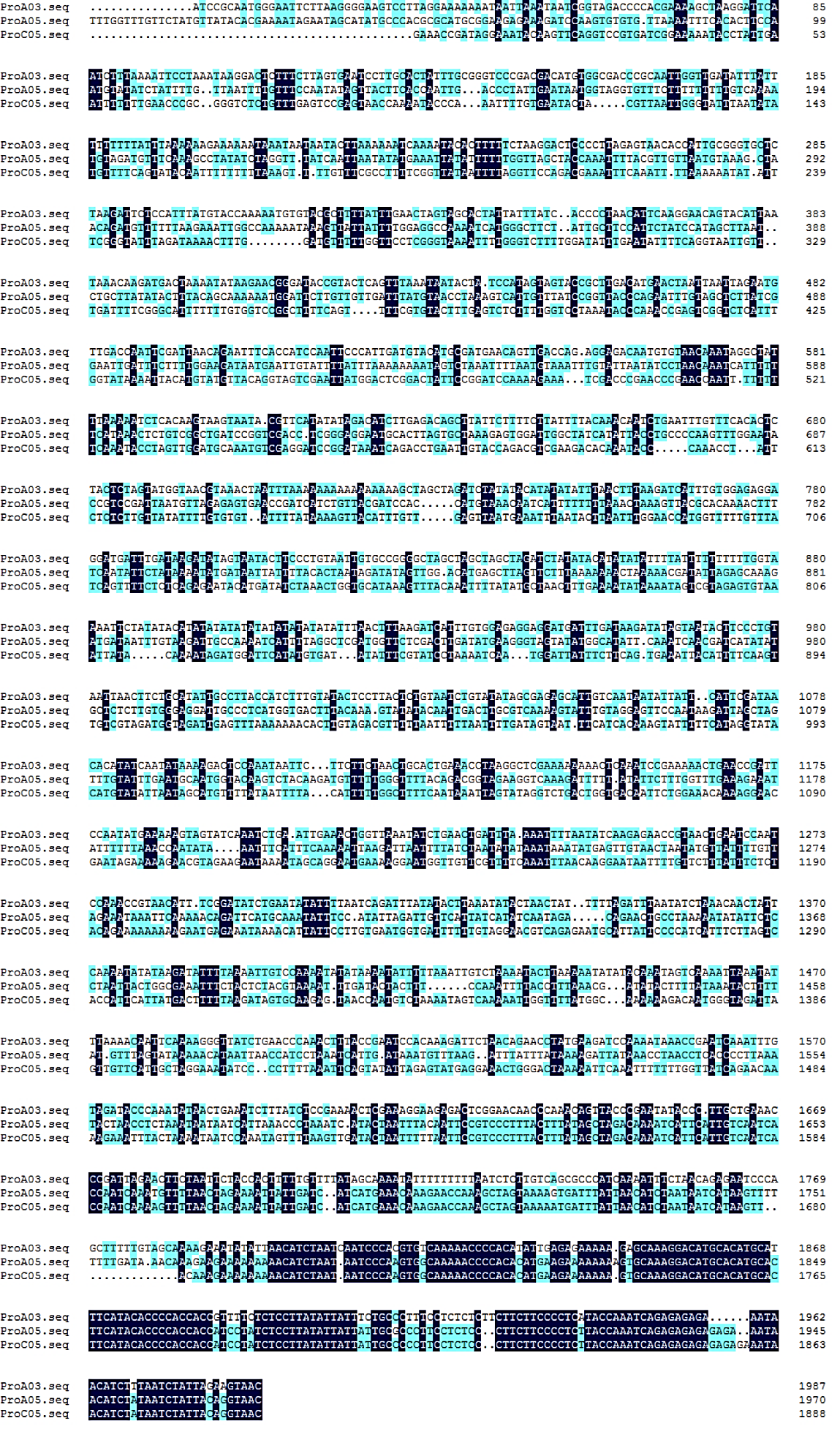


**Supplemental Figure 9.** **Sequence alignment of the three *BnaIAA7* promoters.**

**
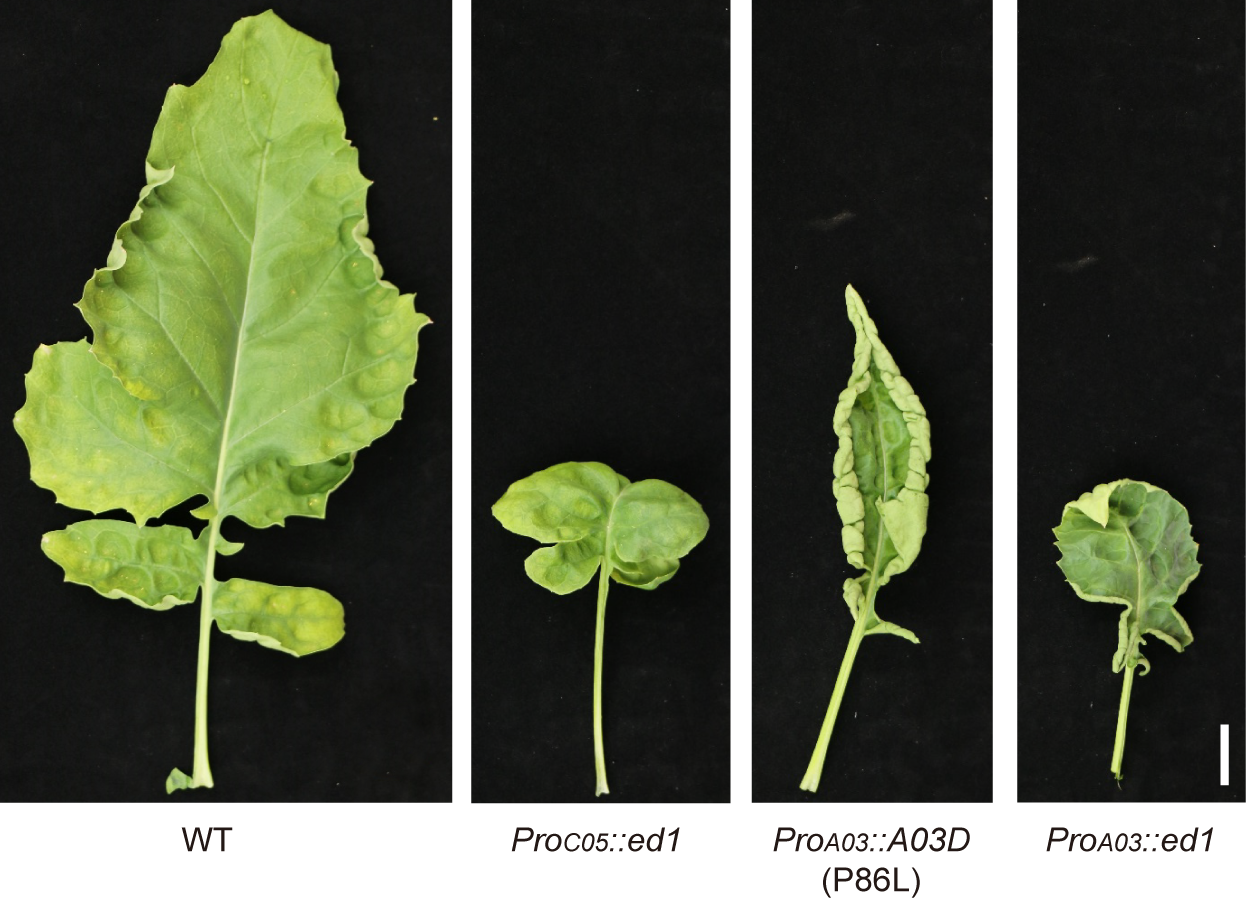
**

**Supplemental Figure 10.** **Different leaf morphologies of *Pro_C05_::ed1*, *Pro_A03_::A03D* (P86L) and *Pro_A03_::ed1* homozygous transgenic plants.**

A03 and C05 indicate *BnaA03*.*IAA7* and *BnaC05*.*IAA7*, respectively. Bar = 2 cm.
